# Health-related quality of life in patients with vestibular schwannoma managed with observation, stereotactic radiosurgery or microsurgery: a systematic review and single-arm meta-analysis

**DOI:** 10.1007/s00415-026-13730-3

**Published:** 2026-03-07

**Authors:** Ineke M. J. Pruijn, Frédérique R. L. M. Welie, Wietske Kievit, Henricus P. M. Kunst

**Affiliations:** 1 Department of Otorhinolaryngology and Head and Neck Surgery-Research Institute for Medical Innovation, Radboud University Medical Center, P.O. Box 9101, 6525 EX Nijmegen, The Netherlands; 2 Dutch Academic Alliance Skull Base Pathology, Radboud University Medical Center, Maastricht University Medical Center+, Nijmegen/Maastricht, The Netherlands; 3 Department of Otorhinolaryngology and Head and Neck Surgery, Maastricht University Medical Center, Maastricht, The Netherlands; 4 Radboud Institute for Medical Innovation, Science Department IQ Health, Radboud University Medical Center, Nijmegen, The Netherlands

**Keywords:** Vestibular schwannoma, Quality of life, Patient-reported outcome measures, Treatment outcome, Systematic review

## Abstract

**Background:**

Health-related quality of life (HRQoL) is a key outcome in the management of vestibular schwannoma (VS). Although wait-and-scan (W&S), stereotactic radiosurgery (SRS), and microsurgery (MS) are established management strategies, their comparative effects on HRQoL remain unclear. This systematic review and meta-analysis aimed to synthesize HRQoL outcomes using the Penn Acoustic Neuroma Quality of Life (PANQOL) questionnaire and to pool PANQOL scores for W&S, SRS, and MS.

**Methods:**

A systematic search of PubMed and Embase was conducted up to February 2025. Eligible studies included patients with unilateral sporadic VS managed with W&S, SRS, or MS, with HRQoL assessed by PANQOL at least one year after diagnosis or intervention. Pooled mean PANQOL scores were calculated using single-arm meta-analyses. Minimal clinically important differences (MCIDs) were applied to assess relevance.

**Results:**

16 studies including 3745 patients were analyzed. The pooled PANQOL total score was 69.1 (95% CI 66.0–72.2) for W&S (*n* = 1,430), 66.9 (95% CI 62.7–71.2) for SRS (*n* = 864), and 61.3 (95% CI 57.2–65.4) for MS (*n* = 1451). Across domains, scores were the lowest for hearing and energy and the highest for facial function and anxiety. None of the between-strategy differences in total or domain scores exceeded established MCID thresholds. Substantial heterogeneity was present across all analyses (*I*^2^ > 75%).

**Conclusion:**

Patients with unilateral VS report broadly comparable HRQoL following W&S, SRS, or MS. Although numerical differences in PANQOL scores exist, they are not clinically meaningful. The observed heterogeneity highlights the need for standardized, prospective studies and international collaboration to better inform patient-centered decision-making.

**Supplementary Information:**

The online version contains supplementary material available at 10.1007/s00415-026-13730-3.

## Introduction

Quality of life (QoL) has gained an increasing importance in the field of vestibular schwannoma (VS), and as such, has become of paramount importance in the decision-making and evaluation of management strategies. Vestibular schwannomas are the most prevalent tumors in the cerebellopontine angle with an incidence rate of 34 VS/million/year [1]. Patients may be faced with unilateral hearing loss, tinnitus, and balance problems resulting from VS [2]. However, the majority of patients may also suffer from fatigue, lack of energy, anxiety, dizziness, or headaches [2–5]. Both these disease-specific and more general symptoms, either caused by the vestibular schwannoma or initiated management, result in limitations and restrictions in daily life activities and societal participation. This ultimately leads to a deterioration of the health-related quality of life (HRQoL).

Management strategies for VS include a conservative wait-and-scan (W&S) strategy with serial imaging and regular follow-up, or active treatment with either stereotactic radiosurgery (SRS) or microsurgery (MS). Given the benign nature of VS and the generally small- to medium-sized tumors at presentation, there has been increasing attention to management-related morbidity, prompting several studies evaluating the impact of different treatment modalities on HRQoL. However, research in this area has been heterogeneous and sometimes contradictory, with studies drawing differing conclusions on the effects of the same management strategy [6–13].

To provide an overall assessment of management impact on QoL, Gauden et al. [14] published a systematic review in 2011, concluding that methodological limitations—including study design and small sample sizes—precluded firm conclusions. At that time, HRQoL in VS patients was primarily assessed using generic instruments, such as the 36-item Short Form Questionnaire (SF-36) and the Glasgow Benefit Inventory (GBI). In 2010, the disease-specific Penn Acoustic Neuroma Quality of Life (PANQOL) questionnaire was introduced [15], providing a more targeted assessment of patient-reported outcomes. The PANQOL captures the unique functional and psychosocial challenges faced by VS patients—including hearing, balance, facial function, and anxiety—and is more sensitive to clinically meaningful changes than generic instruments. Using the PANQOL, studies can ensure consistency in outcome measurement, enhance comparability across treatment modalities, and allow for more precise evaluation of management-related HRQoL effects.

Building on this methodological advancement, Papatsoutsos et al. [16] published an updated systematic review in 2018, extending the work of Gauden et al. [14], summarizing the literature on the impact of management strategies on HRQoL up to 2016. Their findings indicated that VS diagnosis itself was associated with reduced HRQoL, which further deteriorated after microsurgery and stereotactic radiotherapy [16].

It is important to recognize, however, that the previous evaluations have some limitations. While in the time of Gauden et al. there were no studies yet evaluating HRQoL with the disease-specific PANQOL, Papatsoutos et al. searched the literature up to 2016, missing out on the long-term evaluations of HRQoL using the PANQOL. Additionally, their systematic review included studies that reported changes in HRQoL either through visual indicators (e.g., directional arrows in figures) or numerical values, without providing the original baseline or follow-up measurements [17]. Moreover, several studies on HRQoL were conducted in highly specific patient groups [18, 19], making a comparison of treatment strategies and the generalizability problematic.

Though research on HRQoL and patient-reported outcomes evaluating treatments in patients with VS has taken flight in the past years, the question remains which management modality preserves the highest HRQoL [20]. To aid patient counseling, enhance shared decision-making and evaluation of HRQoL outcomes, the main objective of this systematic review and meta-analysis was to pool PANQOL HRQoL outcomes in patients with VS managed with W&S, SRS or MS and secondary to compare and evaluate the differences.

## Materials and methods

This systematic review was performed and reported according to the Preferred Reporting Items for Systematic Reviews and Meta-Analysis (PRISMA) and registered at the International Prospective Register of Systematic Reviews (PROSPERO) under number CRD420250651347.

### Search strategy

A systematic search of literature in PubMed and Embase was conducted up to February 10, 2025. The search was performed with a restriction on publication date including studies from 2010 onwards as the PANQOL was introduced by Shaffer et al. in 2010. A librarian assisted in drafting and pretesting the search strategy in PubMed which was adapted to the other database. The search strategy comprised the key elements “vestibular schwannoma” or “acoustic neuroma” and equivalents combined with “health-related quality of life” or “quality of life” or “PANQOL” or synonyms. Reference lists of eligible studies were reviewed for potential inclusion. Appendix 1 lists the full electronic search strategy.

### Eligibility criteria

We included all original, peer-reviewed studies evaluating at least one management strategy (W&S, SRS or MS) in patients with unilateral sporadic VS, either as investigated population or subpopulation reporting on PANQOL scores as an outcome (total and domain scores). The majority of the patients had to be evaluated with a per protocol analysis. Follow-up time had to be at least one year after diagnosis for W&S management and at least one year after SRS or MS treatment. Articles needed to have full-text availability and be written in English or Dutch. Exclusion criteria were inclusion of patients with neurofibromatosis type II, inclusion of patients with salvage treatment or a combination of treatments, literature or systematic reviews, meta-analyses, case reports, comments, letters to the editor and books. Studies from which the PANQOL data were not extractable from text, tables or supplementary files were also excluded.

### Study selection

Two reviewers (IP and FW) independently screened all titles and abstracts for eligibility using the Rayyan platform [21]. Discordance was reconciled with mutual agreement or, if necessary, discussed with a third reviewer (HK) until consensus was reached. The same reviewers (IP and FW) independently assessed all eligible full texts for inclusion and excluded studies with reason, with disagreements resolved by consensus. In cases where multiple studies originated from the same research group and described overlapping patient populations, the publication with the highest number of patients was included.

### Outcome

The primary outcome measure was the PANQOL total score for each management strategy. Secondary outcome measures included the PANQOL subdomain scores for *Anxiety, Balance, Energy, Face, General, Hearing*, *and Pain*. The PANQOL consists of 26 items across these seven domains, each scored on a 0–100 scale, with higher scores indicating better HRQoL. The PANQOL total score represents the mean of the seven subdomain scores and similarly ranges from 0 to 100. Although the PANQOL was developed as a disease-specific instrument for patients with vestibular schwannoma (VS), data from a Dutch reference sample without VS demonstrated a mean total score of 81.8 (SD 12.6), reflecting that even individuals without VS may experience mild hearing loss, tinnitus, or balance issues [4]. Outcomes were interpreted using the minimal clinically important differences (MCID) defined as the smallest change in a patient-reported outcome that patients perceive as meaningful and clinically relevant [22]. For the PANQOL, established MCID thresholds are 12.5 for the total score, 16.4 for Anxiety, 14 for Balance, 16 for Energy, 25 for Face, 12.9 for General, 13.1 for Hearing, and 20.6 for Pain [22]. These thresholds facilitate the distinction between statistically significant changes and those that are clinically meaningful from the patient’s perspective.

### Data extraction

For each management strategy, main study characteristics were extracted from each article on first author, year of publication, country of study conduction, study design, study period, number of patients, tumor size, mean follow-up time, and type of SRS or MS. Additionally, the PANQOL outcomes (means and standard deviations) were extracted from the data. Attempts were made to contact corresponding authors of included studies to obtain essential data. There was no imputation for missing data. Means and standard deviations were calculated from medians and interquartile ranges if necessary, and pooled means and standard deviations were calculated if there were no combined outcomes available in subgroup analysis i.e., separate reporting on men vs. women [23].

### Quality assessment

All included studies were either single-arm or nonrandomized comparative designs. Therefore, a critical appraisal was conducted using the Risk Of Bias In Nonrandomized Studies—of Interventions (ROBINS-I) assessment tool. This assessment evaluated seven domains: bias due to confounding, selection of participants, classification of interventions, deviations from intended interventions, missing data, measurement of outcomes, and selection of the reported results. The overall risk of bias was rated as high when these domains were not adequately addressed or reported, and low when sufficient methodological detail was provided. No studies were excluded based on the results of this appraisal.

### Statistical analysis

Pooled results on PANQOL total score and PANQOL domain scores per treatment strategy are shown in a single-arm meta-analysis using forest plots with means and confidence intervals. The proportion of variation across studies owing to heterogeneity rather than chance was assessed with the *I*^2^ statistic. Statistical analysis was performed with R version 4.3.1 (R Foundation for Statistical Computing).

## Results

### Study selection

The systematic search strategy identified 973 potentially eligible records. After removal of duplicates, 614 unique articles were screened based on title and abstract. Of these, 66 articles were selected for full-text review, ultimately resulting in the inclusion of 16 studies. [4, 6, 13, 24–36]. A manual search of the reference lists of included studies did not identify any additional eligible publications.

**Fig. 1:**
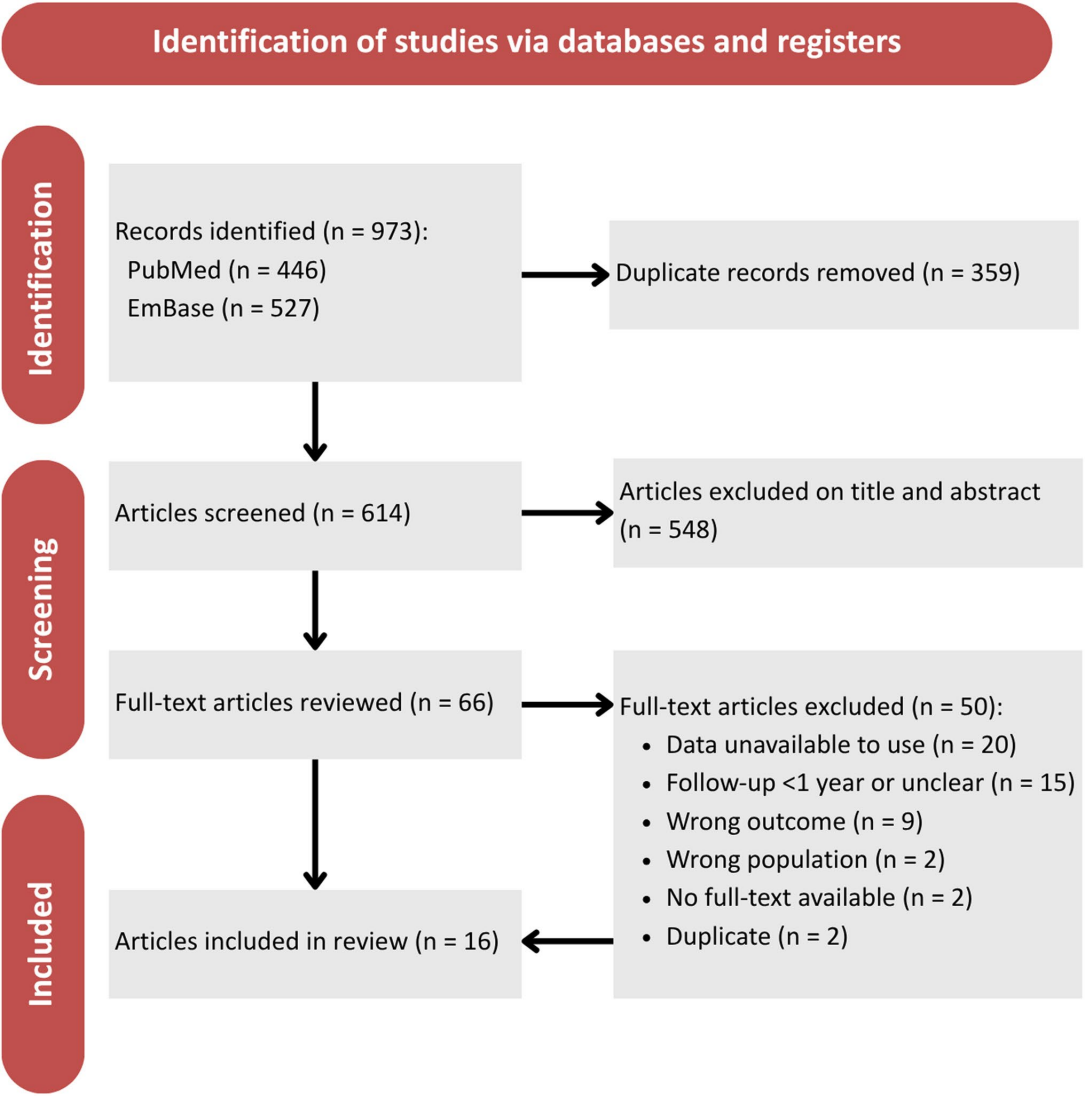
PRISMA flow chart

All included studies were either single-arm or nonrandomized comparative designs, which is why a single-arm meta-analysis was performed for each management strategy, enabling pooling of treatment-specific outcomes without relying on assumptions required for indirect comparisons. Two studies were identified with overlapping patient populations, identical follow-up and PANQOL outcomes. Although the most recent publication by Link et al. appeared in 2018 [37], the earlier study by Carlson et al. (2015) [6] included one additional patient in the MS 20–30 mm CPA group (*n* = 144 vs. *n* = 143). For this reason, Carlson et al. [6] was included in the single-arm meta-analysis, while Link et al. was excluded [37]. The study selection process is summarized in the PRISMA flow diagram (Fig. 1). Detailed study characteristics and outcome data are provided in Appendix 3 and Appendix 4.

### Risk of bias

Of the 16 included studies, 9 studies were categorized as having a moderate risk of bias [4, 6, 13, 25–27, 32, 34, 36] and 7 studies were deemed at serious risk of bias [24, 28–31, 33, 35], primarily due to concerns with missing data and selection of reported results. No studies were excluded based on their bias assessment. See Appendix 2 for the detailed bias assessment.

### Wait-and-scan

12 predominantly cross-sectional studies were included in the single-arm meta-analysis for the W&S strategy. Study characteristics are summarized in Appendix 3. In total, PANQOL data from 1430 patients were pooled, with sample sizes ranging from 13 to 303 participants per study. The mean time from diagnosis to PANQOL assessment ranged between 1.28 and 12.4 years. Tumor size was reported either in centimeters [6, 25–27], or using the Koos classification system [4, 30–32, 36]. Overall, most tumors were smaller than 20 mm or classified as Koos grades I–III.

**Fig. 2:**
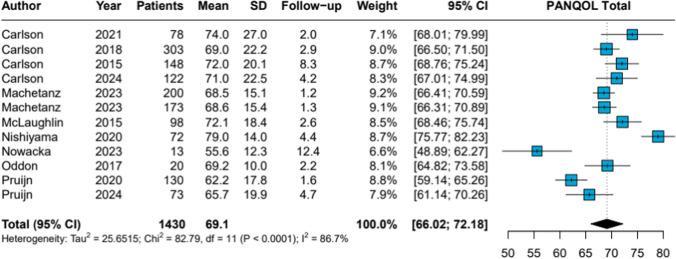
Wait-and-Scan Forest Plot PANQOL Total

The meta-analysis yielded a pooled PANQOL total score of 69.1 (95% CI 66.02–72.18), accompanied by high heterogeneity (*I*^2^ = 86.7%, *p* < 0.0001). The corresponding forest plot is presented in Fig. 2. Pooled subdomain scores were: Anxiety 73.0, Balance 70.0, Energy 67.4, Face 85.8, General 59.2, Hearing 56.2, and Pain 74.5 (Table 1; Forest plots PANQOL subdomain scores in Appendix 5).

**Table 1:** Mean PANQOL scores from single-arm meta-analysis

	W&S	SRS	MS	W&S vs. SRS	W&S vs. MS	SRS vs. MS	MCID
PANQOL anxiety	73.0	73.9	69.1	− 0.9	3.9	4.8	16.4
PANQOL balance	70.0	61.0	58.2	9.0	11.8	2.8	14.0
PANQOL energy	67.4	60.7	58.1	6.7	9.3	2.6	16.0
PANQOL face	85.8	83.5	70.6	2.3	15.2	12.9	25.0
PANQOL general	59.2	59.8	62.3	− 0.6	− 3.1	− 2.5	12.9
PANQOL hearing	56.2	48.9	50.4	7.3	5.8	− 1.5	13.1
PANQOL pain	74.5	72.6	62.0	1.9	12.5	10.6	20.6
PANQOL total	69.1	66.9	61.3	2.2	7.8	5.6	12.5

### Stereotactic radiosurgery

Nine studies were included in the single-arm meta-analysis of SRS, originating from Ireland [24], the USA [6, 13, 25–27], New Zealand [33], and the Netherlands [4, 36]. Sample sizes ranged from 12 to 247 patients, resulting in a total of 864 individuals. Mean follow-up durations varied from 1.1 to 12.4 years. Tumor sizes ranged from a few millimeters to ≥ 40 mm, corresponding to Koos grades I through IV. The most common type of SRS was the Gamma Knife in five out of nine studies [4, 13, 24, 26, 36]. The other four did not report on the type of SRS.

**Fig. 3:**
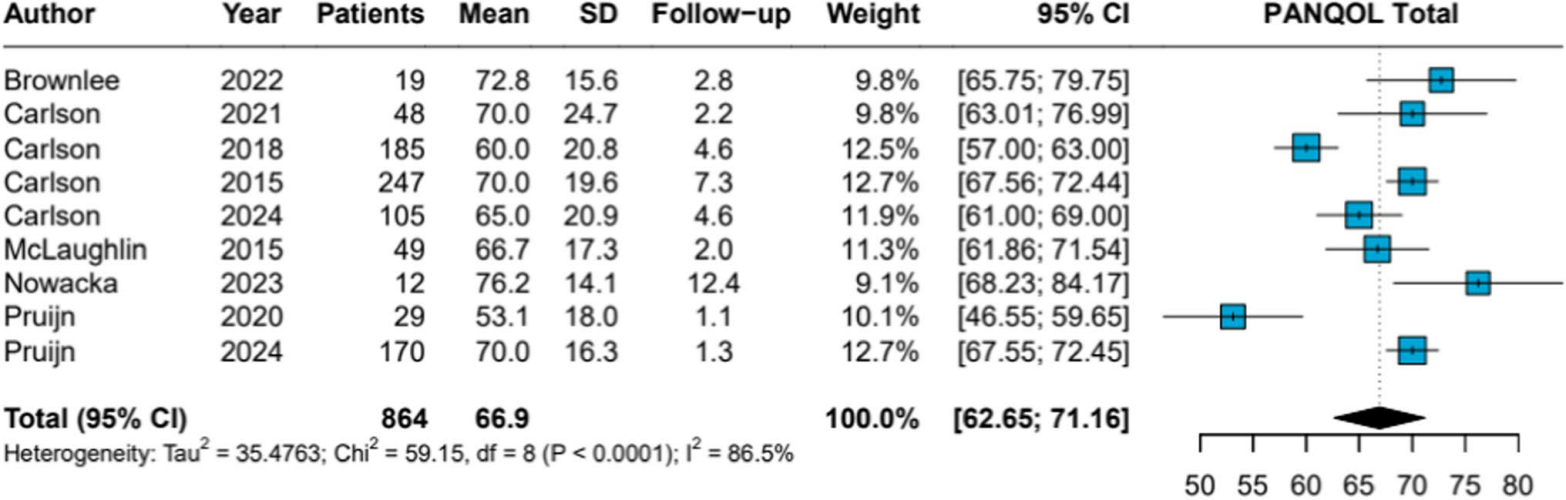
Stereotactic Radiosurgery Forest Plot PANQOL Total

The pooled PANQOL total score following SRS was 66.9 (95% CI 62.65–71.16), with high heterogeneity (*I*^2^ = 86.5%, *p* < 0.0001) (Fig. 3). Pooled subdomain scores were: Anxiety 73.9, Balance 61.0, Energy 60.7, Face 83.5, General 59.8, Hearing 48.9, and Pain 72.6 (Table 1; Forest plots PANQOL subdomain scores in Appendix 6).

### Microsurgery

12 studies were included in the single-arm meta-analysis of microsurgical treatment, encompassing 1451 patients. Most underwent treatment using the retrosigmoid approach, which enables hearing preservation [4, 26, 29–31, 35]. PANQOL outcomes were assessed after a mean follow-up ranging from 1 to 12.4 years. Sample sizes varied from 15 to 507 participants per study. Tumor size distribution is detailed in Appendix 3; however, the majority of patients underwent microsurgery for tumors measuring 10–30 mm or classified as Koos grades III–IV.

**Fig. 4:**
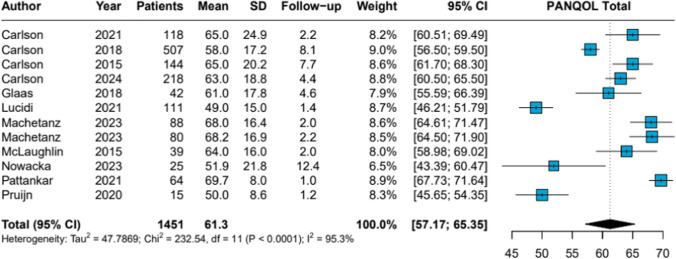
MMicrosurgery Forest Plot PANQOL Total

The pooled PANQOL total score for microsurgical treatment was 61.3 (95% CI 57.17–65.35), with high heterogeneity (*I*^2^ = 95.3%, *p* < 0.0001) (Fig. 4). Pooled subdomain scores were: Anxiety 69.1, Balance 58.2, Energy 58.1, Face 70.6, General 62.3, Hearing 50.4, and Pain 62.0 (Table 1; Forest plots PANQOL subdomain scores in Appendix 7).

No pooled PANQOL total or subdomain score differences between the three management strategies (W&S vs. SRS, W&S vs. MS, or SRS vs. MS) exceeded the MCID (Table 1).

## Discussion

This systematic review and single-arm meta-analysis pooled PANQOL HRQoL scores for patients with unilateral VS managed with W&S, SRS or MS. Overall, patients managed with W&S had a pooled PANQOL total score of 69.1, those managed with SRS scored 66.9, and patients undergoing MS had a pooled PANQOL total score of 61.3. Importantly, differences in total and subdomain PANQOL scores across W&S, SRS, and MS did not exceed the established MCIDs, indicating no clinically meaningful differences in HRQoL and suggesting that outcomes are broadly comparable across treatment modalities.

However, these findings should be considered in the wider context of tumor management and clinical decision-making, where HRQoL outcomes are only one of several important factors. At first glance, it may appear counterintuitive that W&S yields HRQoL outcomes comparable to those of active interventions, such as SRS and MS. However, this should not be interpreted as a lack of therapeutic benefit of active treatment. PANQOL primarily captures symptom burden and functional impact rather than tumor control. Moreover, treatment decisions should not be based solely on HRQoL scores; rather, they must integrate the effectiveness of each management strategy in terms of tumor control, the risks associated with each treatment, including the risk of mortality, treatment-related morbidities, and the potential long-term impact on HRQoL. A comprehensive evaluation of these factors is essential to ensure that patients receive the most appropriate care. Given the complexity inherent in these decisions, shared decision-making is crucial. Clinicians must engage in thorough, transparent, and individualized counseling with patients, providing them with a clear understanding of the potential benefits and risks of each treatment option. This collaborative process is vital for aligning treatment decisions with the patient’s values, preferences, and clinical circumstances, thereby optimizing the selection of the most suitable therapeutic approach.

Nevertheless, the interpretation of pooled PANQOL scores requires caution, given the substantial heterogeneity observed across studies, with *I*^2^ values exceeding 75% in all analyses. This heterogeneity is likely explained by several factors. First, many included studies had a cross-sectional or retrospective design, often lacking power calculations, and failing to report the timing or rationale for initiating treatment. These designs also precluded baseline PANQOL scores and the opportunity to evaluate the change in PANQOL scores during follow-up following W&S, SRS, or MS. Second, variability in patient populations—including tumor size, Koos classification, age, comorbidities, and overall health status—and reporting those may have affected both baseline HRQoL and the impact of treatment. Third, patient and clinician preferences, as well as geographic differences in treatment approaches, likely influenced management choice and outcomes [38]. Finally, differences in response rates, influenced by whether questionnaires were administered in-hospital, online, or via post, may have introduced further variability in reported outcomes [39, 40].

Taken together, these sources of heterogeneity highlight the challenges of synthesizing HRQoL outcomes across diverse study designs and populations. Despite this heterogeneity, pooling of PANQOL scores was methodologically warranted using a random-effects model, which accounts for both within- and between-study variability. This approach allows synthesis of treatment-specific PANQOL outcomes, integrating variability in patient populations, tumor characteristics, and management delivery—including surgical approach, radiosurgery protocols, and follow-up schedules—thereby providing pooled estimates that meaningfully reflect the impact of each management strategy on HRQoL despite the observed heterogeneity.

In addition to this variability within the included literature, several methodological considerations also limited the number of studies eligible for inclusion in the meta-analysis. The V-REX study [41], for example, which is a RCT, was excluded because it used an intention-to-treat (ITT) analysis, which is particularly useful in reflecting real-world clinical scenarios. In clinical practice, patients often switch between treatments or discontinue their assigned therapy, and ITT analysis captures this variability by including all patients as initially assigned, regardless of whether they complete the treatment as planned. This allows for a more realistic estimate of the overall effectiveness of the treatments. However, in the context of this meta-analysis, we sought to isolate the effects of the treatments as per protocol, to better understand the specific outcomes of each treatment modality, and to minimize confounding from therapy switching or discontinuation. As nearly half of the patients in the V-REX study (44%) switched from W&S to active treatment, the HRQoL results were difficult to interpret in the context of actual management strategies when viewed through the lens of ITT. Furthermore, multiple studies with larger cohorts and longer follow-up periods could not be included because they did not report, but only visualized, PANQOL scores, and attempts to obtain these data from the authors were unsuccessful. Studies that lacked measures of variability (SD, IQR) or reported PANQOL outcomes stratified according to other factors (for example, hearing) were similarly excluded, further reducing the available evidence base.

A further limitation of the included studies is the absence of PANQOL baseline scores, precluding meaningful assessment of within-group changes in HRQoL over time for each management strategy. Without baseline measurements, it remains unclear whether reported follow-up scores reflect true stability, improvement, or deterioration relative to pretreatment status. In addition, limited reporting on patients lost to follow-up—particularly the reasons for non-response—raises concerns about attrition bias. In patients who may experience greater morbidity as a result of their VS or treatment modality, HRQoL may be more severely impacted, potentially leading to non-completion of questionnaires. Therefore, these patients, who may represent the most clinically relevant cases, are often underrepresented in the final HRQoL scores. This could introduce bias and limit the generalizability of the findings.

Beyond these practical exclusions, there remains a more fundamental question regarding the ability of the current PANQOL instrument to capture true differences between management strategies. One could argue whether there actually are differences to be measured between management strategies, given that pooled PANQOL HRQoL scores show only small variations that do not exceed the MCID. This raises the question whether these differences are truly negligible or simply not captured due to limitations in the PANQOL instrument. The PANQOL equally weights all seven domains in calculating the total score, despite evidence that certain domains—such as energy, anxiety, pain, and balance—contribute more strongly to overall HRQoL than others like hearing or tinnitus [3, 4]. This uniform weighting may obscure clinically meaningful differences between patient groups or treatment modalities. The recently developed Vestibular Schwannoma Quality of Life (VSQOL) index [42], which includes additional domains, such as cognitive function, treatment satisfaction, and broader pain assessment, has been designed to capture a more comprehensive and nuanced view of HRQoL. Given its enhanced sensitivity and psychometric robustness, the VSQOL may be better suited to detect (subtle) clinically relevant differences in patient-reported outcomes that the PANQOL cannot fully reveal. Future studies employing the VSQOL could therefore determine whether subtle but clinically relevant differences exist between treatment strategies, ultimately supporting more informed, patient-centered decision-making.

This naturally leads to the broader issue of how the field can generate higher-level evidence in future. Ultimately, the highest level of evidence regarding HRQoL outcomes in patients with vestibular schwannoma would be obtained from meta-analyses of RCTs or rigorously controlled cohort studies directly comparing management strategies. However, such studies are exceedingly scarce, reflecting both ethical and practical challenges in randomizing patients to distinct treatment modalities in this population. An alternative approach could be a network meta-analysis, in which studies comparing different pairs of interventions—for example, W&S versus SRS and SRS versus MS—are synthesized to enable indirect comparisons across all three strategies. While this method has theoretical appeal, its validity depends on sufficient overlap in baseline patient, tumor and HRQoL characteristics across studies to satisfy the transitivity assumption. Moreover, network meta-analysis fundamentally relies on randomized comparative trials using a common comparator to construct the network, as randomization ensures that patients with similar baseline characteristics could theoretically have been allocated to any treatment arm across studies. Observational studies, including matched cohort designs, do not fully satisfy this assumption and therefore may introduce residual confounding if incorporated into the network. In the context of VS HRQoL research, such comparability is generally lacking, hence the high heterogeneity in this meta-analysis, limiting the interpretability of indirect comparisons. Given these constraints, single-arm meta-analyses remain the most feasible and methodologically defensible approach to synthesize existing data, providing valuable insights into patient-reported outcomes across treatment modalities while transparently acknowledging the limitations imposed by heterogeneity and study design.

Looking forward, strengthening the evidence base will require coordinated efforts to enhance data quality and comparability across studies. Future research should prioritize standardized collection and reporting of HRQoL outcomes, including the consistent use of validated instruments such as the PANQOL or VSQOL at baseline and during follow-up, complete reporting of measures of variability, and stratification according to clinically relevant factors, such as tumor size, treatment indication, and baseline patient, tumor, and HRQoL characteristics. Establishing a national database and fostering international collaboration would facilitate larger and more representative datasets. This, in turn, would enable individual patient data (IPD) meta-analyses, which are widely considered the most methodologically rigorous approach to evidence synthesis. By allowing direct access to raw, patient-level data across centers, IPD meta-analyses enable uniform data harmonization, adjustment for confounding variables, and exploration of subgroup effects that are not possible with aggregate data alone. Such an approach would substantially improve the validity and interpretability of comparative effectiveness research in vestibular schwannoma. Ultimately, the goal of these efforts is to generate high-quality, generalizable evidence that informs shared decision-making, helping patients to weigh the potential impact of different management strategies on their quality of life and to select the treatment that best aligns with their individual preferences and clinical circumstances.

## Conclusion

In summary, this systematic review and single-arm meta-analysis demonstrates that patients with unilateral VS managed with W&S, SRS, or MS report broadly comparable HRQoL outcomes, with differences in pooled PANQOL scores not exceeding the corresponding MCIDs. These findings suggest that, from a HRQoL perspective, no management strategy confers a clear advantage over the others. However, these findings should be considered in the wider context of tumor management and clinical decision-making, where HRQoL outcomes are only one of several important factors. Nevertheless, substantial heterogeneity, methodological limitations, and incomplete reporting highlight the need for more rigorous and standardized research. Establishing national databases and fostering international collaboration will be essential to generate sufficiently large and representative datasets, enabling more robust analyses and subgroup explorations. Ultimately, the overarching aim of such efforts is to provide high-quality, generalizable evidence that can support patients and clinicians in making well-informed, individualized treatment decisions.

## Supplementary Information

Below is the link to the electronic supplementary material.

Supplementary file1 (DOCX 14 kb)

Supplementary file2 (DOCX 18 kb)

Supplementary file3 (DOCX 33 kb)

Supplementary file4 (DOCX 42 kb)

Supplementary file5 (PDF 186 kb)

Supplementary file6 (PDF 102 kb)

Supplementary file7 (PDF 140 kb)

## Data Availability

Data will be made available upon reasonable request.

## References

[1] Reznitsky M, Petersen M, West N, Stangerup SE, Caye-Thomasen P (2019) Epidemiology of vestibular schwannomas - prospective 40-year data from an unselected national cohort. Clin Epidemiol 11:981–986. 10.2147/clep.S218670 31807080 10.2147/CLEP.S218670 PMC6850685

[2] Broomfield SJ, O’Donoghue GM (2016) Self-reported symptoms and patient experience: a British Acoustic Neuroma Association survey. Br J Neurosurg 30(3):294–301. 10.3109/02688697.2015.1071323 26523744 10.3109/02688697.2015.1071323

[3] Carlson ML, Tveiten OV, Driscoll CL et al (2015) What drives quality of life in patients with sporadic vestibular schwannoma? Laryngoscope 125(7):1697–1702. 10.1002/lary.25110 25546382 10.1002/lary.25110

[4] Pruijn IMJ, Kievit W, Hentschel MA, Mulder JJS, Kunst HPM (2021) What determines quality of life in patients with vestibular schwannoma? Clin Otolaryngol 46(2):412–420. 10.1111/coa.13691 33326685 10.1111/coa.13691 PMC7986908

[5] Pruijn IMJ, van Heemskerken P, Kunst HPM, Tummers M, Kievit W (2023) Patient-preferred outcomes in patients with vestibular schwannoma: a qualitative content analysis of symptoms, side effects and their impact on health-related quality of life. Qual Life Res 32(10):2887–2897. 10.1007/s11136-023-03433-x 37258945 10.1007/s11136-023-03433-x PMC10474211

[6] Carlson ML, Tveiten OV, Driscoll CL et al (2015) Long-term quality of life in patients with vestibular schwannoma: an international multicenter cross-sectional study comparing microsurgery, stereotactic radiosurgery, observation, and nontumor controls. J Neurosurg 122(4):833–842. 10.3171/2014.11.Jns14594 25555165 10.3171/2014.11.JNS14594

[7] Regis J, Carron R, Park MC et al (2010) Wait-and-see strategy compared with proactive Gamma Knife surgery in patients with intracanalicular vestibular schwannomas. J Neurosurg 113(Suppl):105–111. 10.3171/2010.8.Gks101058 21121792 10.3171/2010.8.GKS101058

[8] Regis J, Pellet W, Delsanti C et al (2002) Functional outcome after Gamma Knife surgery or microsurgery for vestibular schwannomas. J Neurosurg 97(5):1091–1100. 10.3171/jns.2002.97.5.1091 12450031 10.3171/jns.2002.97.5.1091

[9] Di Maio S, Akagami R (2009) Prospective comparison of quality of life before and after observation, radiation, or surgery for vestibular schwannomas. J Neurosurg 111(4):855–862. 10.3171/2008.10.Jns081014 19301957 10.3171/2008.10.JNS081014

[10] Sandooram D, Grunfeld EA, McKinney C, Gleeson MJ (2004) Quality of life following microsurgery, radiosurgery and conservative management for unilateral vestibular schwannoma. Clin Otolaryngol Allied Sci 29(6):621–627. 10.1111/j.1365-2273.2004.00881.x 15533149 10.1111/j.1365-2273.2004.00881.x

[11] Myrseth E, Moller P, Pedersen PH, Lund-Johansen M (2009) Vestibular schwannoma: surgery or Gamma Knife radiosurgery? A prospective, nonrandomized study. Neurosurgery 64(4):654–661. 10.1227/01.NEU.0000340684.60443.55. (**discussion 661-3**) 19197222 10.1227/01.NEU.0000340684.60443.55

[12] Pollock BE, Driscoll CL, Foote RL et al (2006) Patient outcomes after vestibular schwannoma management: a prospective comparison of microsurgical resection and stereotactic radiosurgery. Neurosurgery 59(1):77–85. 10.1227/01.Neu.0000219217.14930.14. (**discussion 77-85**) 16823303 10.1227/01.NEU.0000219217.14930.14

[13] McLaughlin EJ, Bigelow DC, Lee JY, Ruckenstein MJ (2015) Quality of life in acoustic neuroma patients. Otol Neurotol 36(4):653–656. 10.1097/mao.0000000000000674 25473957 10.1097/MAO.0000000000000674

[14] Gauden A, Weir P, Hawthorne G, Kaye A (2011) Systematic review of quality of life in the management of vestibular schwannoma. J Clin Neurosci 18(12):1573–1584. 10.1016/j.jocn.2011.05.009 22014598 10.1016/j.jocn.2011.05.009

[15] Shaffer BT, Cohen MS, Bigelow DC, Ruckenstein MJ (2010) Validation of a disease-specific quality-of-life instrument for acoustic neuroma: the Penn Acoustic Neuroma Quality-of-Life Scale. Laryngoscope 120(8):1646–1654. 10.1002/lary.20988 20641085 10.1002/lary.20988

[16] Papatsoutsos E, Spielmann PM (2018) Self-evaluated quality of life and functional outcomes after microsurgery, stereotactic radiation or observation-only for vestibular schwannoma of the adult patient: a systematic review. Otol Neurotol 39(2):232–241. 10.1097/MAO.0000000000001664 29315189 10.1097/MAO.0000000000001664

[17] Sandooram D, Hornigold R, Grunfeld B, Thomas N, Kitchen ND, Gleeson M (2010) The effect of observation versus microsurgical excision on quality of life in unilateral vestibular schwannoma: a prospective study. Skull Base 20(1):47–54. 10.1055/s-0029-1242985 20592858 10.1055/s-0029-1242985 PMC2853062

[18] Godefroy WP, Hastan D, van der Mey AG (2007) Translabyrinthine surgery for disabling vertigo in vestibular schwannoma patients. Clin Otolaryngol 32(3):167–172. 10.1111/j.1365-2273.2007.01427.x 17550503 10.1111/j.1365-2273.2007.01427.x

[19] Sun D, Shi Z, Li P, Shi S, Cai Y (2015) Psychological status and quality of life in acoustic neuroma patients with facial palsy after microsurgery: a 1-year postoperative follow-up study. Acta Neurol Belg 115(3):311–316. 10.1007/s13760-014-0382-z 25344828 10.1007/s13760-014-0382-z

[20] Goldbrunner R, Weller M, Regis J et al (2020) EANO guideline on the diagnosis and treatment of vestibular schwannoma. Neuro Oncol 22(1):31–45. 10.1093/neuonc/noz153 31504802 10.1093/neuonc/noz153 PMC6954440

[21] Ouzzani M, Hammady H, Fedorowicz Z, Elmagarmid A (2016) Rayyan-a web and mobile app for systematic reviews. Syst Rev 5(1):210. 10.1186/s13643-016-0384-4 27919275 10.1186/s13643-016-0384-4 PMC5139140

[22] Kerezoudis P, Yost KJ, Tombers NM, Celda MP, Carlson ML, Link MJ (2019) Defining the minimal clinically important difference for patients with vestibular schwannoma: are all quality-of-life scores significant? Neurosurgery 85(6):779–785. 10.1093/neuros/nyy467 30395303 10.1093/neuros/nyy467

[23] Wan X, Wang W, Liu J, Tong T (2014) Estimating the sample mean and standard deviation from the sample size, median, range and/or interquartile range. BMC Med Res Methodol 14(1):135. 10.1186/1471-2288-14-135 25524443 10.1186/1471-2288-14-135 PMC4383202

[24] Brownlee N, Wilson C, Curran DB, Wright G, Flannery T, Caldwell SB (2022) Cognitive and psychosocial outcomes following stereotactic radiosurgery for acoustic neuroma. NeuroRehabilitation 50(1):151–159. 10.3233/nre-210106 34957955 10.3233/NRE-210106

[25] Carlson ML, Babajanian EE, Lohse CM, Tombers NM, Link MJ (2024) Long-term prospective quality-of-life outcomes in 445 patients with sporadic vestibular schwannoma. Otol Neurotol 45(10):1167–1171. 10.1097/mao.0000000000004328 39439048 10.1097/MAO.0000000000004328

[26] Carlson ML, Barnes JH, Nassiri A et al (2021) Prospective study of disease-specific quality-of-life in sporadic vestibular schwannoma comparing observation, radiosurgery, and microsurgery. Otol Neurotol 42(2):e199–e208. 10.1097/mao.0000000000002863 33177408 10.1097/MAO.0000000000002863

[27] Carlson ML, Tombers NM, Kerezoudis P, Celda MP, Lohse CM, Link MJ (2018) Quality of life within the first 6 months of vestibular schwannoma diagnosis with implications for patient counseling. Otol Neurotol 39(10):e1129–e1136. 10.1097/mao.0000000000001999 30239440 10.1097/MAO.0000000000001999

[28] Glaas MF, Schäfer R, Jansen P et al (2018) Quality of life after translabyrinthine vestibular schwannoma resection-reliability of the German PANQOL questionnaire. Otol Neurotol 39(6):e481–e488. 10.1097/mao.0000000000001819 29889791 10.1097/MAO.0000000000001819

[29] Lucidi D, Fabbris C, Cerullo R et al (2022) Quality of life in vestibular schwannoma: a comparison of three surgical techniques. Eur Arch Otorhinolaryngol 279(4):1795–1803. 10.1007/s00405-021-06855-w 33963915 10.1007/s00405-021-06855-w

[30] Machetanz K, Lee L, Wang SS, Tatagiba M, Naros G (2023) Trading mental and physical health in vestibular schwannoma treatment decision. Front Oncol 13:1152833. 10.3389/fonc.2023.1152833 37434979 10.3389/fonc.2023.1152833 PMC10332305

[31] Machetanz K, Wang SS, Oberle L, Tatagiba M, Naros G (2023) Sex differences in vestibular schwannoma. Cancers (Basel). 10.3390/cancers15174365 37686642 10.3390/cancers15174365 PMC10486905

[32] Nishiyama T, Oishi N, Kojima T et al (2020) Validation and multidimensional analysis of the Japanese Penn acoustic neuroma quality-of-life scale. Laryngoscope 130(12):2885–2890. 10.1002/lary.28488 31922264 10.1002/lary.28488

[33] Nowacka A, Barker-Collo S, Miles A, Ben-Harosh L (2023) The effect of symptomatology and mental wellbeing on quality of life in people with acoustic neuroma. J Clin Neurosci 116:1–7. 10.1016/j.jocn.2023.08.005 37597328 10.1016/j.jocn.2023.08.005

[34] Oddon PA, Montava M, Salburgo F, Collin M, Vercasson C, Lavieille JP (2017) Conservative treatment of vestibular schwannoma: growth and Penn Acoustic Neuroma Quality of Life scale in French language. Acta Otorhinolaryngol Ital 37(4):320–327. 10.14639/0392-100x-1094. (**Trattamento conservativo degli schwannomi vestibolari: accrescimento e Penn Acoustic Quality of Life scale in lingua francese**) 28872162 10.14639/0392-100X-1094 PMC5584105

[35] Pattankar S, Churi O, Misra BK (2021) Quality of life in patients of unilateral vestibular schwannoma treated with microsurgery: a South-Asian tertiary care hospital experience. J Clin Neurosci 89:264–270. 10.1016/j.jocn.2021.04.034 34119279 10.1016/j.jocn.2021.04.034

[36] Pruijn IMJ, Parmaksiz M, Verheul JB, Mulder JJS, Kievit W, Kunst HPM (2024) Health-related quality of life in patients with a stable or growing vestibular schwannoma managed by wait and scan or stereotactic radiosurgery. Otolaryngol Head Neck Surg 171(3):823–832. 10.1002/ohn.814 38769852 10.1002/ohn.814

[37] Link MJ, Lund-Johansen M, Lohse CM et al (2018) Quality of life in patients with vestibular schwannomas following gross total or less than gross total microsurgical resection: Should we be taking the entire tumor out? Neurosurgery 82(4):541–547. 10.1093/neuros/nyx245 29554375 10.1093/neuros/nyx245

[38] Carlson ML, Glasgow AE, Grossardt BR, Habermann EB, Link MJ (2016) Does where you live influence how your vestibular schwannoma is managed? Examining geographical differences in vestibular schwannoma treatment across the United States. J Neurooncol 129(2):269–279. 10.1007/s11060-016-2170-5 27334903 10.1007/s11060-016-2170-5

[39] Neve OM, van Benthem PPG, Stiggelbout AM, Hensen EF (2021) Response rate of patient reported outcomes: the delivery method matters. BMC Med Res Methodol 21(1):220. 10.1186/s12874-021-01419-2 34686129 10.1186/s12874-021-01419-2 PMC8540148

[40] Prummer CM, Kerezoudis P, Tombers NM, Peris-Celda M, Link MJ, Carlson ML (2019) Influence of selection bias in survey studies derived from a patient-focused organization: A comparison of response data from a single tertiary care center and the Acoustic Neuroma Association. Otol Neurotol 40(4):504–510. 10.1097/mao.0000000000002151 30870367 10.1097/MAO.0000000000002151

[41] Dhayalan D, Tveiten ØV, Finnkirk M et al (2023) Upfront radiosurgery vs a wait-and-scan approach for small- or medium-sized vestibular schwannoma: The V-REX randomized clinical trial. JAMA 330(5):421–431. 10.1001/jama.2023.12222 37526718 10.1001/jama.2023.12222 PMC10394573

[42] Carlson ML, Lohse CM, Link MJ et al (2023) Development and validation of a new disease-specific quality of life instrument for sporadic vestibular schwannoma: the Mayo Clinic Vestibular Schwannoma Quality of Life Index. J Neurosurg 138(4):981–991. 10.3171/2022.7.Jns221104 36057121 10.3171/2022.7.JNS221104

