# Supplementary material for: Health-related quality of life in patients with vestibular schwannoma managed with observation, stereotactic radiosurgery or microsurgery: a systematic review and single-arm meta-analysis
Source: J Neurol. 2026 Mar 7;273(3):187. doi: 10.1007/s00415-026-13730-3 (PMC12967669; doi:10.1007/s00415-026-13730-3)
Supplement: Supplementary file 1 — Supplementary file1 (DOCX 14 kb) [file 415_2026_13730_MOESM1_ESM.docx]

**PubMed**

#1

("neuroma, acoustic"[MeSH Terms:noexp] OR "acoustic neuroma*"[tiab] OR "acoustic schwannoma*"[tiab] OR “schwannoma, acoustic”[tiab] OR "neurinoma of the acoustic nerve*"[tiab] OR "acoustic neurilemoma*"[tiab] OR "vestibular schwannoma*"[tiab] OR “Schwannoma, Vestibular”[tiab] OR "acoustic tumor*"[tiab] OR "acoustic tumour*"[tiab] OR "acoustic neurilemmoma*"[tiab] OR "acoustic neurinoma*"[tiab])

#2

("Quality of Life"[Mesh] OR "quality of life"[tiab] OR life qualit*[tiab] OR living qualit*[tiab] OR "quality of living"[tiab] OR "qol"[tiab] OR "hrql"[tiab] OR "hrqol"[tiab])

#3

(“PANQOL”[Text Word] OR “Penn Acoustic Neuroma Quality Of Life”[Text Word])

(#1 AND #2) OR #3

**Embase**

1. ((exp acoustic neuroma/ OR acoustic nerve neurinoma.ti,ab,kf. OR acoustic nerve tumor*.ti,ab,kf. OR acoustic nerve tumour*.ti,ab,kf. OR acoustic tumor*.ti,ab,kf. OR acoustic tumour*.ti,ab,kf. OR acoustic neurinoma*.ti,ab,kf. OR acoustic neurilemmoma*.ti,ab,kf. OR acoustic neurilemoma*.ti,ab,kf. OR acoustic schwannoma*.ti,ab,kf. OR acusticus neurinoma*.ti,ab,kf. OR auditory nerve neurinoma*.ti,ab,kf. OR vestibular schwannoma*.ti,ab,kf.)
2. (exp quality of life/ OR (life adj1 qualit*).ti,ab,kf. OR quality of life.ti,ab,kf. OR qol.ti,ab,kf. OR hrql.ti,ab,kf. OR hrqol.ti,ab,kf.))
3. (PANQOL.ti,ab,kf. OR Penn Acoustic Neuroma Quality Of Life.ti,ab,kf.)
4. 2 OR 3
5. 1 AND 4
