# Supplementary material for: Health-related quality of life in patients with vestibular schwannoma managed with observation, stereotactic radiosurgery or microsurgery: a systematic review and single-arm meta-analysis
Source: J Neurol. 2026 Mar 7;273(3):187. doi: 10.1007/s00415-026-13730-3 (PMC12967669; doi:10.1007/s00415-026-13730-3)
Supplement: Supplementary file 2 — Supplementary file2 (DOCX 18 kb) [file 415_2026_13730_MOESM2_ESM.docx]

| Study | Bias Due To Confounding | Bias In Classification Of Interventions | Bias In Selection Of Participants Into The Study | Bias Due To Deviations From Intended Interventions | Bias Due To Missing Data | Bias In Measurements Of The Outcome | Bias In Selection Of Reported Result | Overall |
| --- | --- | --- | --- | --- | --- | --- | --- | --- |
| Brownlee 2022 | Moderate | Low | Moderate | Moderate | Low | Low | Moderate | Serious |
| Carlson 2015 | Moderate | Low | Moderate | Moderate | Low | Low | Low | Moderate |
| Carlson 2018 | Moderate | Low | Moderate | Moderate | Low | Low | Low | Moderate |
| Carlson 2021 | Moderate | Low | Moderate | Moderate | Low | Low | Low | Moderate |
| Carlson 2024 | Moderate | Low | Moderate | Moderate | Low | Low | Low | Moderate |
| Glaas 2018 | Moderate | Low | Moderate | Moderate | Moderate | Low | Low | Serious |
| Lucidi 2022 | Moderate | Low | Moderate | Moderate | Moderate | Low | Low | Serious |
| Machetanz 2023 | Moderate | Low | Moderate | Moderate | Moderate | Low | Low | Serious |
| Machetanz 2023 | Moderate | Low | Moderate | Moderate | Moderate | Low | Low | Serious |
| Mclaughlin 2015 | Moderate | Low | Moderate | Moderate | Low | Low | Low | Moderate |
| Nishiyama 2020 | Moderate | Low | Moderate | Moderate | Low | Low | Low | Moderate |
| Nowacka 2023 | Serious | Moderate | Moderate | Moderate | Serious | Low | Low | Serious |
| Oddon 2017 | Moderate | Low | Moderate | Moderate | Low | Low | Low | Moderate |
| Pattankar 2021 | Moderate | Low | Moderate | Moderate | Low | Low | Moderate | Serious |
| Pruijn 2020 | Moderate | Low | Moderate | Moderate | Low | Low | Low | Moderate |
| Pruijn 2024 | Moderate | Low | Moderate | Moderate | Low | Low | Low | Moderate |
