# Supplementary material for: Health-related quality of life in patients with vestibular schwannoma managed with observation, stereotactic radiosurgery or microsurgery: a systematic review and single-arm meta-analysis
Source: J Neurol. 2026 Mar 7;273(3):187. doi: 10.1007/s00415-026-13730-3 (PMC12967669; doi:10.1007/s00415-026-13730-3)
Supplement: Supplementary file 3 — Supplementary file3 (DOCX 33 kb) [file 415_2026_13730_MOESM3_ESM.docx]

|  | Author | Year | Country | Study design | Study period | Patients (n) | Tumor size | Follow-up  (Mean ± SD) (yr) |
| --- | --- | --- | --- | --- | --- | --- | --- | --- |
| 1 | **Carlson** | 2021 | USA | Prospective | Not reported | 78 | Baseline tumor size in cm 0-0.9: 54 (69%) 1-1.9: 23 (29%) 2-2.9: 1 (1%) 3-3.9: 0 4+: 0 | 2.0 (1.2) |
| 2 | **Carlson** | 2018 | USA | Cross-sectional | Not reported | 303 | Baseline tumor size in cm 0-0.9: 145 (56%) 1-1.9: 90 (35%) 2-2.9: 17 (7%) 3-3.9: 1 (0.3%) 4+: 4 (2%) | 2.9 (3.9) |
| 3 | **Carlson** | 2015 | USA + Norway | Cross-sectional | 1998-2008 | 148 | Intracanalicular: 86 (60%) 0-9mm CPA: 30 (21%) 10-19mm CPA: 26 (18%) 20-30mm CPA: 1 (1%) | 8.3 |
| 4 | **Carlson** | 2024 | USA | Prospective | 2014-2023 | 122 | Baseline tumor size in cm 0-0.9: 68 (60%) 1-1.9: 36 (32%) 2-2.9: 8 (7%) 3+: 1 (1%) | 4.2 (2.4) |
| 5 | **Machetanz** | 2023 | Germany | Cross-sectional | 2019-2022 | 200 | Koos I: 42 (21%) Koos II: 67 (33.5%) Koos III: 59 (29.5%) Koos IV: 32 (16%) | 1.2 (2.2) |
| 6 | **Machetanz** | 2023 | Germany | Cross-sectional | 2019-2021 | 173 | Koos I: 35 (20.2%) Koos II: 59 (34.1%) Koos III: 55 (31.8%) Koos IV: 24 (13.8%) | 1.3 (2.2) |
| 7 | **McLaughlin** | 2015 | USA | Retrospective | 2010-2013 | 98 | Size (maximum dimension mm): 8 (SD 4.7) Extracanalicular component: 23% | 2.6 |
| 8 | **Nishiyama** | 2020 | Japan | Cross-sectional | 2016-2017 | 72 | Koos I: 39 (54.2%) Koos II-IV: 33 (45.8%) | 4.4 (0.4) |
| 9 | **Nowacka** | 2023 | New Zealand | Cross-sectional | 2022 | 13 | Not reported | 12.4 (8.5) |
| 10 | **Oddon** | 2017 | France | Retrospective | 2002-2012 | 20 | Not reported | 2.15 (1.4) |
| 11 | **Pruijn** | 2020 | Netherlands | Cross-sectional | 2014-2017 | 130 | Koos I: 68 (52.3%) Koos II: 31 (23.8%) Koos III: 17 (13.1%) Koos IV: 14 (10.8%) | 1.6 (0.8) |
| 12 | **Pruijn** | 2024 | Netherlands | Cohort | 2014-2022 | 73 | Koos I: 36 (49.3%) Koos II: 20 (27.4%) Koos III: 5 (6.8%) Koos IV: 12 (16.4%) | 4.7 (0.7) |

|  | Author | Year | Country | Study design | Study period | Patients (n) | Tumor size | Follow-up (Mean ± SD) (yr) | Type of SRS |
| --- | --- | --- | --- | --- | --- | --- | --- | --- | --- |
| 1 | **Brownlee** | 2022 | Ireland | Cross-sectional | 2017-2021 | 19 | Not reported | 2.8 (1.4) | Gamma Knife |
| 2 | **Carlson** | 2021 | USA | Prospective | Not reported | 48 | Baseline tumor size in cm 0-0.9: 19 (40%) 1-1.9: 23 (48%) 2-2.9: 6 (13%) 3-3.9: 0 4+: 0 | 2.2 (1.2) | Gamma Knife |
| 3 | **Carlson** | 2018 | USA | Cross-sectional | Not reported | 185 | Baseline tumor size in cm 0-0.9: 42 (25%) 1-1.9: 77 (47%) 2-2.9: 38 (23%) 3-3.9: 6 (4%) 4+: 2 (1%) | 4.6 (4.5) | Not reported |
| 4 | **Carlson** | 2015 | USA + Norway | Cross-sectional | 1998-2008 | 247 | Intracanalicular: 35 (14%) 0-9mm CPA: 65 (26%) 10-19mm CPA: 128 (52%) 20-30mm CPA: 19 (8%) | 7.3 | Not reported |
| 5 | **Carlson** | 2024 | USA | Prospective | 2014-2023 | 105 | Baseline tumor size in cm 0-0.9: 34 (36%) 1-1.9: 46 (49%) 2-2.9: 13 (14%) 3+: 1 (1%) | 4.6 (2.0) | Not reported |
| 6 | **McLaughlin** | 2015 | USA | Retrospective | 2010-2013 | 49 | Size (maximum dimension mm): 18 (SD 5.9) Extracanalicular component: 51% | 2 | Gamma Knife |
| 7 | **Nowacka** | 2023 | New Zealand | Cross-sectional | 2022 | 12 | Not reported | 12.4 (8.5) | Not reported |
| 8 | **Pruijn** | 2020 | Netherlands | Cohort | 2014-2017 | 29 | Koos I: 5 (17.2%) Koos II: 9 (31.0%) Koos III: 7 (24.1%) Koos IV: 8 (27.6%) | 1.1 (0.7) | Gamma Knife |
| 9 | **Pruijn** | 2024 | Netherlands | Cohort | 2014-2022 | 170 | Koos I: 55 (32.4%) Koos II: 73 (42.9%) Koos III: 20 (11.8%) Koos IV: 22 (12.9%) | 1.3 (0.7) | Gamma Knife |

|  | Author | Year | Country | Study design | Study period | Patients (n) | Tumor size | Follow-up (Mean ± SD) (yr) | Type of Microsurgery |
| --- | --- | --- | --- | --- | --- | --- | --- | --- | --- |
| 1 | **Carlson** | 2021 | USA | Prospective | Not reported | 118 | Baseline tumor size in cm 0-0.9: 23 (19%) 1-1.9: 45 (38%) 2-2.9: 34 (29%) 3-3.9: 12 (10%) 4+: 4 (3%) | 2.2 (1.1) | Retrosigmoid: 71 (60%) Translabyrinthine: 45 (38%) Middle Fossa: 1 (1%) Transotic: 1 (1%) |
| 2 | **Carlson** | 2018 | USA | Cross-sectional | Not reported | 507 | Baseline tumor size in cm 0-0.9: 60 (13%) 1-1.9: 120 (26%) 2-2.9: 131 (28%) 3-3.9: 83 (18%) 4+: 67( 15%) | 8.1 (9.4) | Not reported |
| 3 | **Carlson** | 2015 | USA + Norway | Cross-sectional | 1998-2008 | 144 | Intracanalicular: 33 (23%) 0-9mm CPA: 17 (12%) 10-19mm CPA: 57 (40%) 20-30mm CPA: 37 (26%) | 7.7 | Not reported |
| 4 | **Carlson** | 2024 | USA | Prospective | 2014-2023 | 218 | Baseline tumor size in cm 0-0.9: 34 (17%) 1-1.9: 81 (40%) 2-2.9: 62 (31%) 3+: 26 (13%) | 4.4 (2.3) | Not reported |
| 5 | **Glaas** | 2018 | Germany | Cross-sectional | 2007-2017 | 42 | Not reported | 4.6 | Translabyrinthine: 42 (100%) |
| 6 | **Lucidi** | 2021 | Italy | Retrospective | 2017-2020 | 111 | Koos I: 19 (17%) Koos II: 29 (26%) Koos III: 46 (41%) Koos IV: 17 (16%) | 1.4 (0.8) | Retrosigmoid: 48 (43%) Translabyrinthine: 26 (23%) Transcanal transpromontorial: 37 (33%) |
| 7 | **Machetanz** | 2023 | Germany | Cross-sectional | 2019-2022 | 88 | Koos I: 2 (2.3%) Koos II: 14 (15.9%) Koos III: 36 (40.9%) Koos IV: 36 (40.9%) | 2.0 (2.7) | Retrosigmoid–Transmeatal: 88 (100%) |
| 8 | **Machetanz** | 2023 | Germany | Cross-sectional | 2019-2021 | 80 | Koos I: 1 (1.3%) Koos II: 14 (17.5%) Koos III: 32 (40%) Koos IV: 33 (41.3%) | 2.2 (3) | Retrosigmoid–Transmeatal: 80 (100%) |
| 9 | **McLaughlin** | 2015 | USA | Retrospective | 2010-2013 | 39 | Size (maximum dimension mm): 22 (SD 8.3) Extracanalicular component: 69% | 2 | Not reported |
| 10 | **Nowacka** | 2023 | New Zealand | Cross-sectional | 2022 | 25 | Not reported | 12.4 (8.5) | Not reported |
| 11 | **Pattankar** | 2021 | India | Cross-sectional | 2017-2018 | 64 | Small (≤1 mm): 4 (6.25%) Medium (11-20mm): 13 (20.31%) Moderately large (21-30mm): 18 (28.12%) Large (31-40mm): 24 (37.5%) Giant (>40mm): 5 (7.81%) | 1 | Retrosigmoid: 64 (100%) |
| 12 | **Pruijn** | 2020 | Netherlands | Cohort | 2014-2017 | 15 | Koos I: 2 (13.3%) Koos II: 0 Koos III: 0 Koos IV: 13 (86.7%) | 1.2 (0.4) | Retrosigmoid: 14 (93%) Not reported: 1 (7%) |
