# Supplementary material for: Health-related quality of life in patients with vestibular schwannoma managed with observation, stereotactic radiosurgery or microsurgery: a systematic review and single-arm meta-analysis
Source: J Neurol. 2026 Mar 7;273(3):187. doi: 10.1007/s00415-026-13730-3 (PMC12967669; doi:10.1007/s00415-026-13730-3)
Supplement: Supplementary file 4 — Supplementary file4 (DOCX 42 kb) [file 415_2026_13730_MOESM4_ESM.docx]

|  |  |  | PANQOL | | | | | | | | | | | | | | | |
| --- | --- | --- | --- | --- | --- | --- | --- | --- | --- | --- | --- | --- | --- | --- | --- | --- | --- | --- |
|  | **Author** | **Year** | **Total** | ***SD*** | **Anxiety** | ***SD*** | **Balance** | ***SD*** | **General** | ***SD*** | **Hearing** | ***SD*** | **Energy** | ***SD*** | **Headache** | ***SD*** | **Face** | ***SD*** |
| 1 | **Carlson** | 2021 | **74.0** | *27.0* | **78.0** | *33.8* | **74.0** | *36.0* | **67.0** | *31.5* | **62.0** | *33.8* | **71.0** | *41.0* | **83.0** | *45.1* | **84.0** | *29.3* |
| 2 | **Carlson** | 2018 | **69.0** | *22.2* | **71.0** | *26.6* | **67.0** | *26.6* | **68.0** | *22.2* | **58.0** | *22.2* | **62.0** | *26.6* | **73.0** | *35.5* | **86.0** | *22.2* |
| 3 | **Carlson** | 2015 | **72.0** | *20.1* | **80.0** | *25.5* | **74.0** | *30.0* | **58.0** | *19.5* | **60.0** | *29.8* | **67.0** | *27.2* | **83.0** | *19.4* | **87.0** | *18.7* |
| 4 | **Carlson** | 2024 | **71.0** | *22.5* | **77.0** | *33.8* | **68.0** | *31.0* | **64.0** | *25.4* | **59.0** | *31.0* | **68.0** | *31.0* | **78.0** | *39.4* | **82.0** | *25.4* |
| 5 | **Machetanz** | 2023 | **68.5** | *15.1* | **64.8** | *22.5* | **71.4** | *24.1* | **55.1** | *17.1* | **64.9** | *21.7* | **67.5** | *23.0* | **66.2** | *29.6* | **89.1** | *14.3* |
| 6 | **Machetanz** | 2023 | **68.6** | *15.4* | **66.1** | *22.5* | **70.9** | *24.8* | **55.0** | *17.3* | **64.0** | *22.0* | **67.5** | *23.7* | **66.9** | *29.2* | **89.5** | *14.6* |
| 7 | **McLaughlin** | 2015 | **72.1** | *18.4* | **74.0** | *30.0* | **73.0** | *27.0* | **57.0** | *18.0* | **62.0** | *26.0* | **68.0** | *27.0* | **83.0** | *24.0* | **88.0** | *17.0* |
| 8 | **Nishiyama** | 2020 | **79.0** | *14.0* | **83.0** | *19.0* | **86.0** | *18.0* | **59.0** | *18.0* | **72.0** | *22.0* | **79.0** | *20.0* | **80.0** | *25.0* | **92.0** | *14.0* |
| 9 | **Nowacka** | 2023 | **55.6** | *12.3* | **59.1** | *21.3* | **61.9** | *24.6* | **48.1** | *19.7* | **39.4** | *14.1* | **57.3** | *22.8* | **57.7** | *23.7* | **81.4** | *19.9* |
| 10 | **Oddon** | 2017 | **69.2** | *10.0* | **72.4** | *23.2* | **62.8** | *28.2* | **59.7** | *19.9* | **57.2** | *25.3* | **69.3** | *25.7* | **78.9** | *32.6* | **84.2** | *15.4* |
| 11 | **Pruijn** | 2020 | **62.2** | *17.8* | **71.3** | *23.4* | **60.4** | *25.3* | **56.9** | *22.4* | **36.7** | *20.9* | **63.6** | *24.1* | **68.3** | *30.8* | **77.9** | *22.8* |
| 12 | **Pruijn** | 2024 | **65.7** | *19.9* | **76.2** | *22.0* | **64.5** | *27.6* | **59.8** | *22.1* | **38.2** | *26.5* | **66.3** | *25.6* | **73.3** | *29.4* | **81.5** | *21.0* |

|  |  |  | PANQOL | | | | | | | | | | | | | | | |
| --- | --- | --- | --- | --- | --- | --- | --- | --- | --- | --- | --- | --- | --- | --- | --- | --- | --- | --- |
|  | **Author** | **Year** | **Total** | ***SD*** | **Anxiety** | ***SD*** | **Balance** | ***SD*** | **General** | ***SD*** | **Hearing** | ***SD*** | **Energy** | ***SD*** | **Headache** | ***SD*** | **Face** | ***SD*** |
| 1 | **Brownlee** | 2022 | **72.8** | *15.6* | **73.5** | *20.4* | **72.9** | *20.5* | **68.3** | *21.3* | **63.8** | *22.2* | **67.6** | *23.0* | **77.7** | *28.7* | **85.4** | *18.9* |
| 2 | **Carlson** | 2021 | **70.0** | *24.7* | **78.0** | *35.3* | **65.0** | *33.6* | **63.0** | *28.3* | **57.0** | *31.8* | **62.0** | *35.3* | **80.0** | *38.9* | **87.0** | *26.5* |
| 3 | **Carlson** | 2018 | **60.0** | *20.8* | **66.0** | *27.8* | **52.0** | *24.3* | **61.0** | *20.8* | **42.0** | *27.8* | **49.0** | *27.8* | **65.0** | *34.7* | **82.0** | *24.3* |
| 4 | **Carlson** | 2015 | **70.0** | *19.6* | **81.0** | *18.7* | **68.0** | *28.7* | **60.0** | *18.3* | **57.0** | *25.9* | **67.0** | *19.0* | **76.0** | *19.6* | **86.0** | *19.6* |
| 5 | **Carlson** | 2024 | **65.0** | *20.9* | **71.0** | *31.4* | **64.0** | *31.4* | **59.0** | *23.5* | **48.0** | *28.8* | **58.0** | *31.4* | **71.0** | *36.6* | **83.0** | *26.1* |
| 6 | **McLaughlin** | 2015 | **66.7** | *17.3* | **70.0** | *27.0* | **63.0** | *25.0* | **56.0** | *16.0* | **55.0** | *23.0* | **65.0** | *25.0* | **77.0** | *24.0* | **81.0** | *20.0* |
| 7 | **Nowacka** | 2023 | **76.2** | *14.1* | **84.4** | *20.7* | **67.8** | *24.9* | **71.9** | *20.7* | **59.9** | *19.9* | **74.0** | *21.6* | **83.3** | *16.3* | **88.2** | *15.3* |
| 8 | **Pruijn** | 2020 | **53.1** | *18.0* | **65.5** | *24.8* | **42.5** | *23.0* | **53.8** | *24.1* | **29.0** | *20.9* | **50.5** | *24.5* | **61.4** | *31.5* | **69.6** | *24.0* |
| 9 | **Pruijn** | 2024 | **70.0** | *16.3* | **77.2** | *21.0* | **57.2** | *26.8* | **58.5** | *21.8* | **32.4** | *21.6* | **58.4** | *25.1* | **65.6** | *34.5* | **84.2** | *19.2* |

|  |  |  | PANQOL | | | | | | | | | | | | | | | |
| --- | --- | --- | --- | --- | --- | --- | --- | --- | --- | --- | --- | --- | --- | --- | --- | --- | --- | --- |
|  | **Author** | **Year** | **Total** | ***SD*** | **Anxiety** | ***SD*** | **Balance** | ***SD*** | **General** | ***SD*** | **Hearing** | ***SD*** | **Energy** | ***SD*** | **Headache** | ***SD*** | **Face** | ***SD*** |
| 1 | **Carlson** | 2021 | **65.0** | *24.9* | **76.0** | *36.0* | **60.0** | *33.3* | **66.0** | *27.7* | **54.0** | *33.3* | **59.0** | *36.0* | **60.0** | *41.6* | **78.0** | *24.9* |
| 2 | **Carlson** | 2018 | **58.0** | *17.2* | **68.0** | *28.7* | **53.0** | *23.0* | **67.0** | *17.2* | **43.0** | *23.0* | **51.0** | *23.0* | **57.0** | *23.0* | **65.0** | *23.0* |
| 3 | **Carlson** | 2015 | **65.0** | *20.2* | **77.0** | *19.3* | **62.0** | *18.4* | **64.0** | *18.1* | **52.0** | *16.2* | **65.0** | *29.9* | **64.0** | *22.2* | **76.0** | *33.2* |
| 4 | **Carlson** | 2024 | **63.0** | *18.8* | **74.0** | *30.1* | **58.0** | *30.1* | **66.0** | *22.6* | **49.0** | *26.4* | **57.0** | *30.1* | **60.0** | *33.9* | **74.0** | *26.4* |
| 5 | **Glaas** | 2018 | **61.0** | *17.8* | **70.7** | *21.7* | **52.8** | *26.8* | **51.2** | *21.4* | **50.6** | *23.4* | **59.8** | *23.4* | **70.4** | *25.0* | **71.6** | *25.0* |
| 6 | **Lucidi** | 2021 | **49.0** | *15.0* | **41.0** | *22.0* | **52.0** | *22.0* | **58.0** | *23.0* | **56.0** | *19.0* | **42.0** | *17.0* | **43.0** | *27.0* | **54.0** | *23.0* |
| 7 | **Machetanz** | 2023 | **68.0** | *16.4* | **73.7** | *20.9* | **67.5** | *21.9* | **64.2** | *19.4* | **60.8** | *22.3* | **68.0** | *23.6* | **70.6** | *29.8* | **74.8** | *23.8* |
| 8 | **Machetanz** | 2023 | **68.2** | *16.9* | **73.9** | *21.8* | **67.4** | *22.5* | **63.9** | *19.5* | **60.1** | *22.4* | **68.1** | *23.9* | **70.4** | *31.9* | **75.2** | *23.8* |
| 9 | **McLaughlin** | 2015 | **64.0** | *16.0* | **72.0** | *24.0* | **64.0** | *27.0* | **60.0** | *13.0* | **47.0** | *25.0* | **61.0** | *26.0* | **69.0** | *36.0* | **74.0** | *23.0* |
| 10 | **Nowacka** | 2023 | **51.9** | *21.8* | **69.0** | *24.0* | **42.7** | *24.2* | **59.5** | *25.6* | **42.5** | *25.1* | **48.0** | *31.3* | **50.0** | *34.6* | **60.3** | *31.1* |
| 11 | **Pattankar** | 2021 | **69.7** | *8.0* | **76.3** | *19.3* | **71.8** | *23.7* | **65.8** | *20.5* | **55.7** | *20.0* | **68.9** | *19.2* | **69.0** | *20.9* | **80.5** | *18.9* |
| 12 | **Pruijn** | 2020 | **50.0** | *8.6* | **58.1** | *13.9* | **43.2** | *17.9* | **52.3** | *19.3* | **29.9** | *14.2* | **47.0** | *13.7* | **59.4** | *29.2* | **60.6** | *15.9* |
