# Supplementary figures and images for: Health-related quality of life in patients with vestibular schwannoma managed with observation, stereotactic radiosurgery or microsurgery: a systematic review and single-arm meta-analysis
Source: J Neurol. 2026 Mar 7;273(3):187. doi: 10.1007/s00415-026-13730-3 (PMC12967669; doi:10.1007/s00415-026-13730-3)

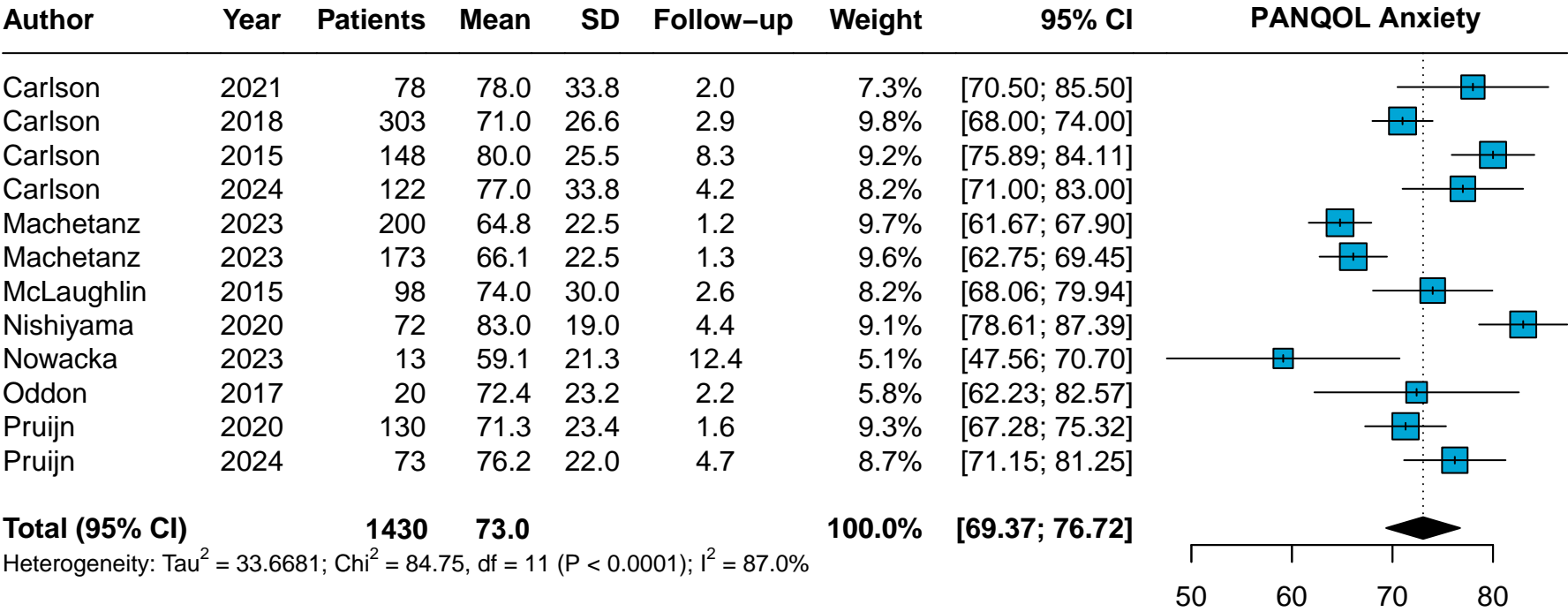

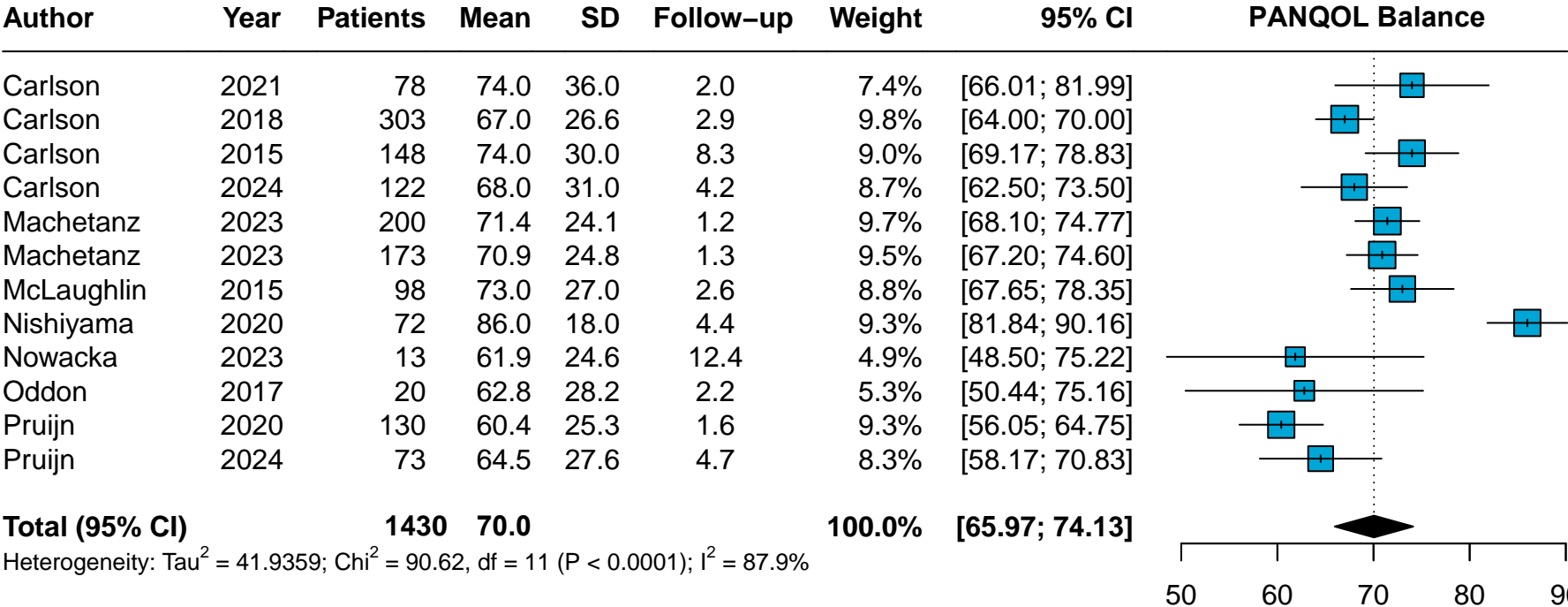

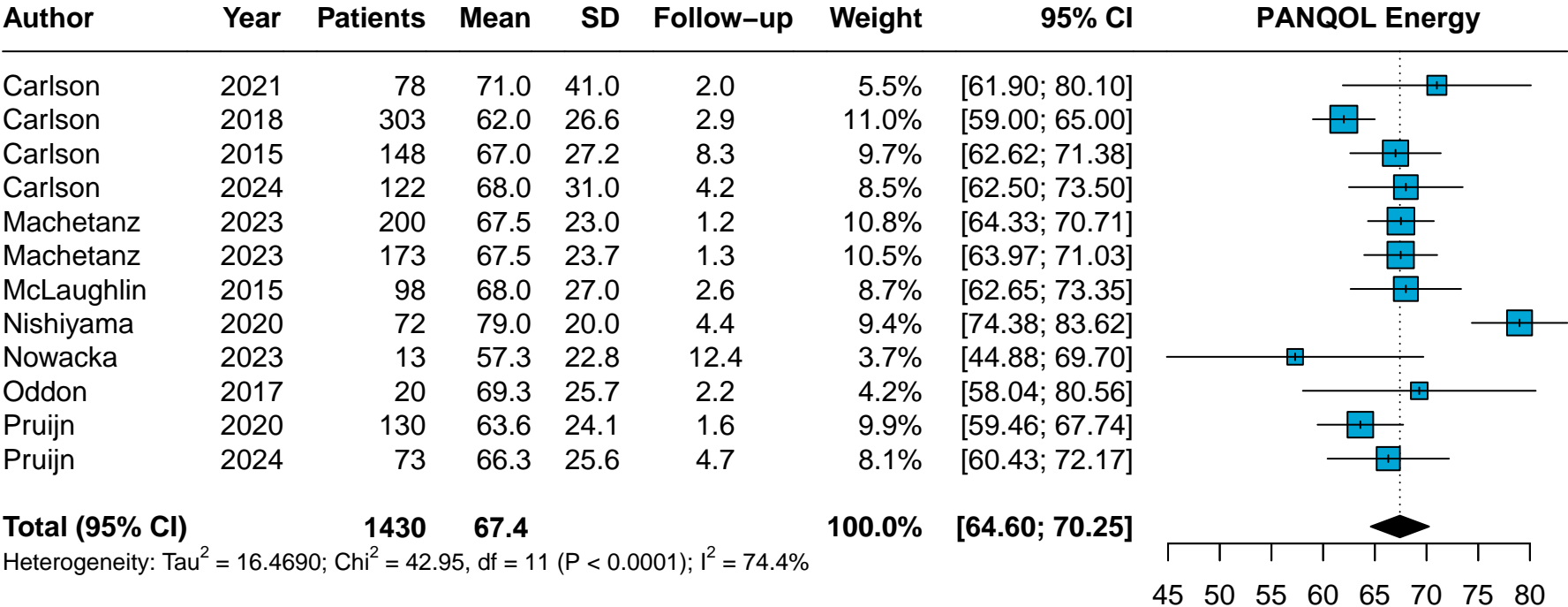

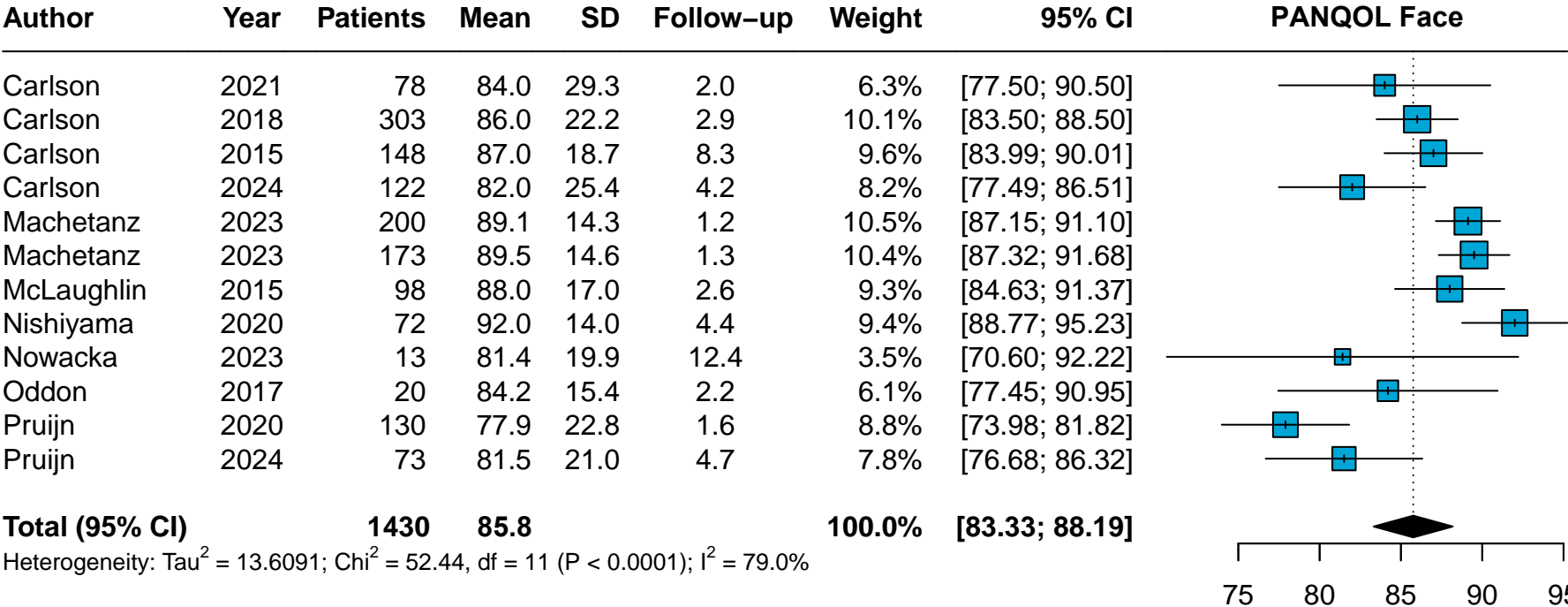

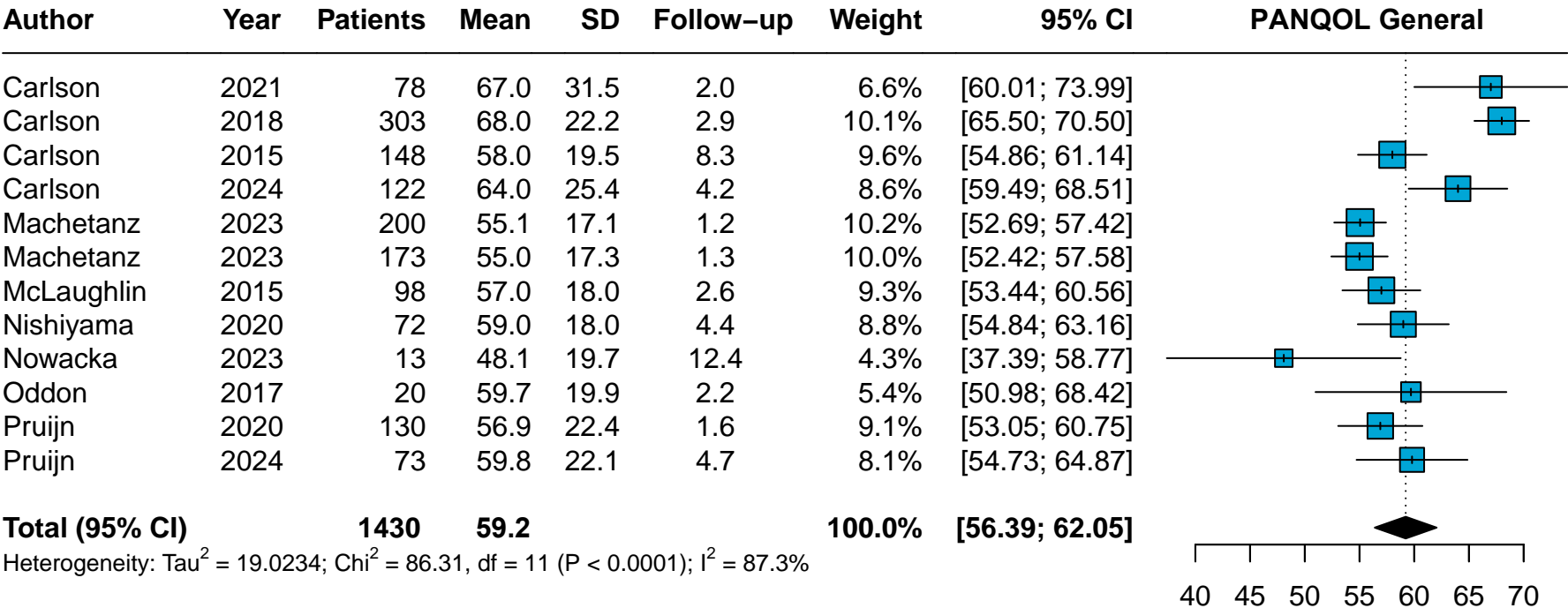

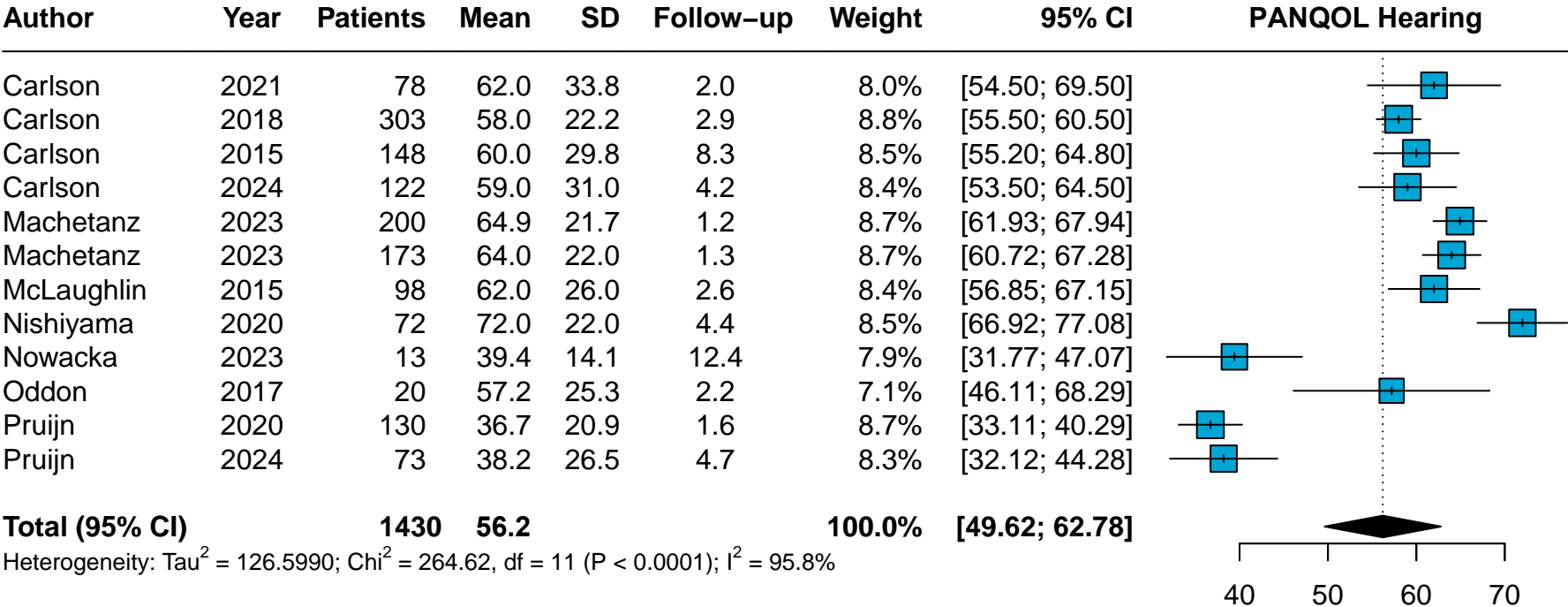

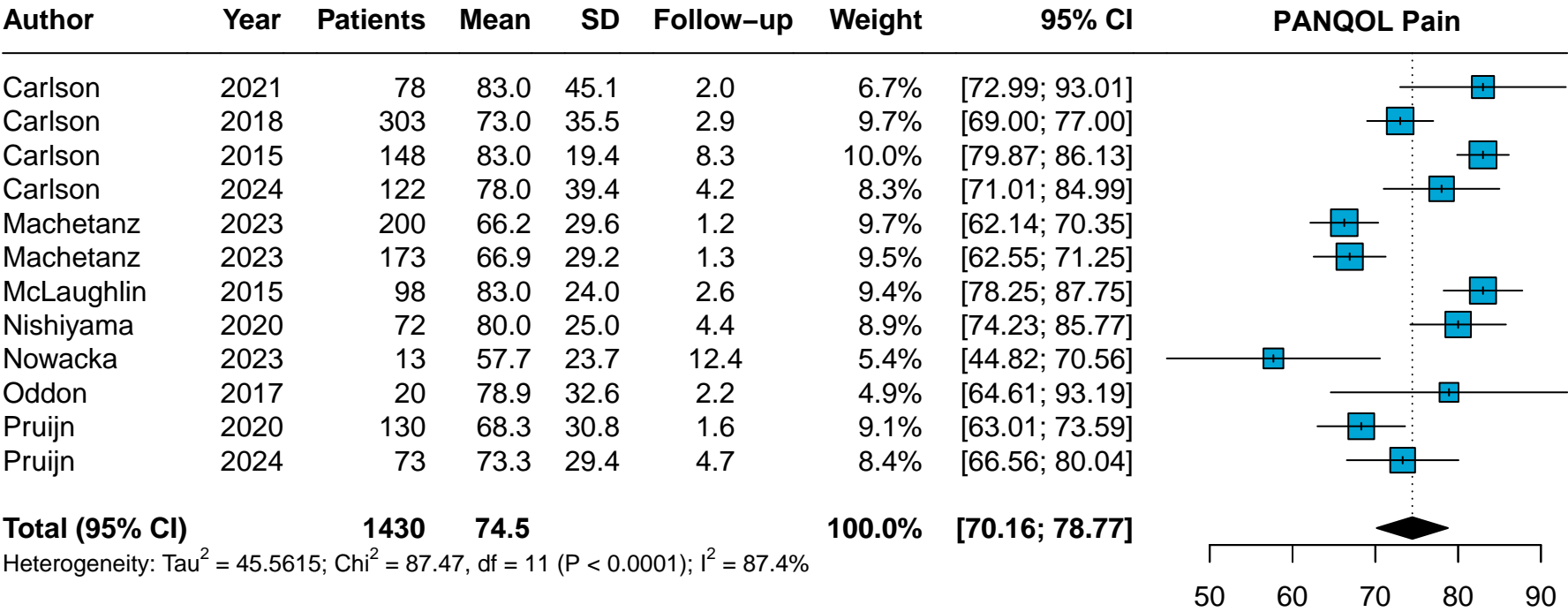

Supplement: Supplementary file 5 — Supplementary file5 (PDF 186 kb) [file 415_2026_13730_MOESM5_ESM.pdf]

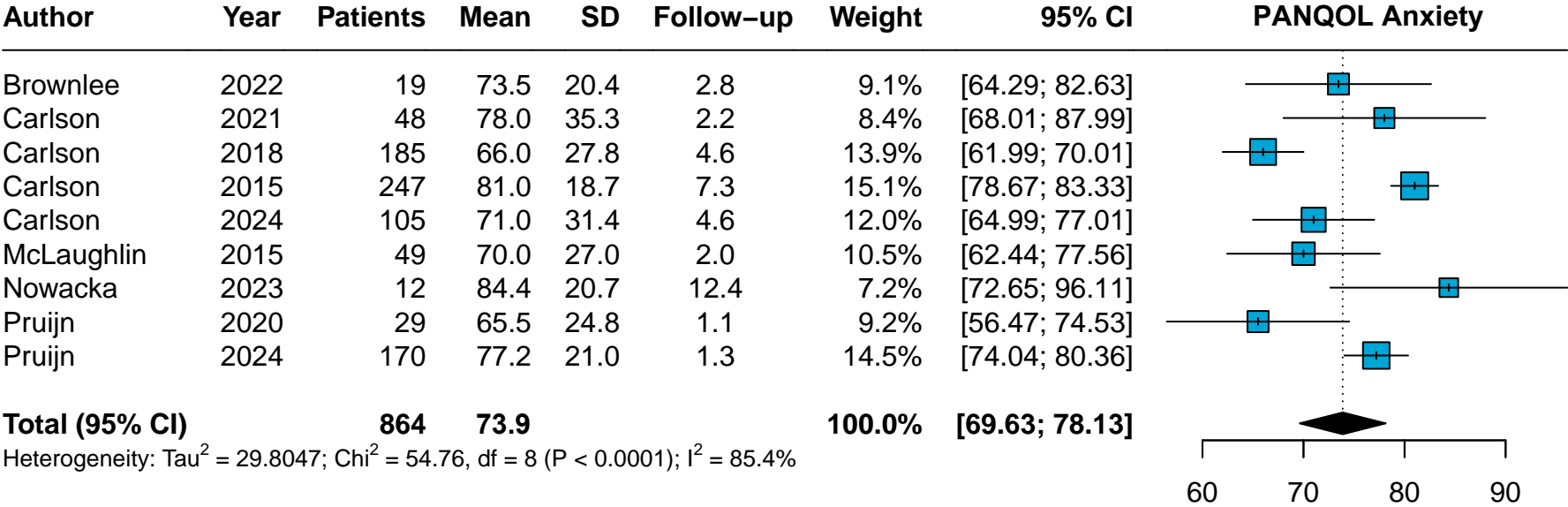

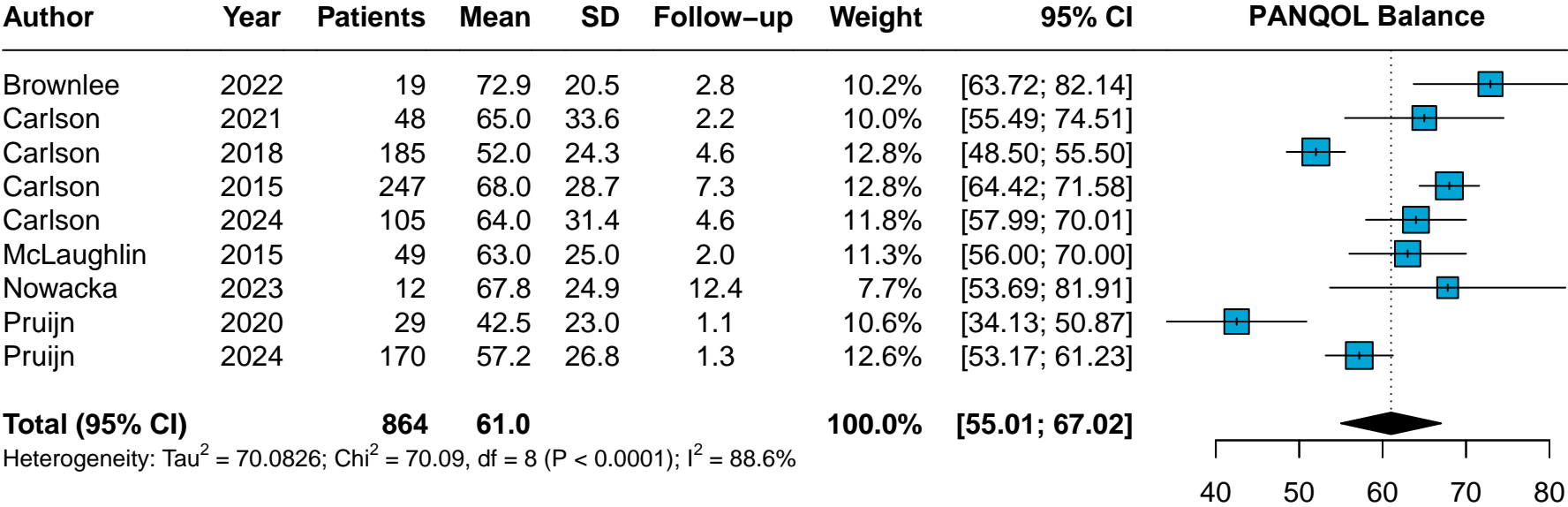

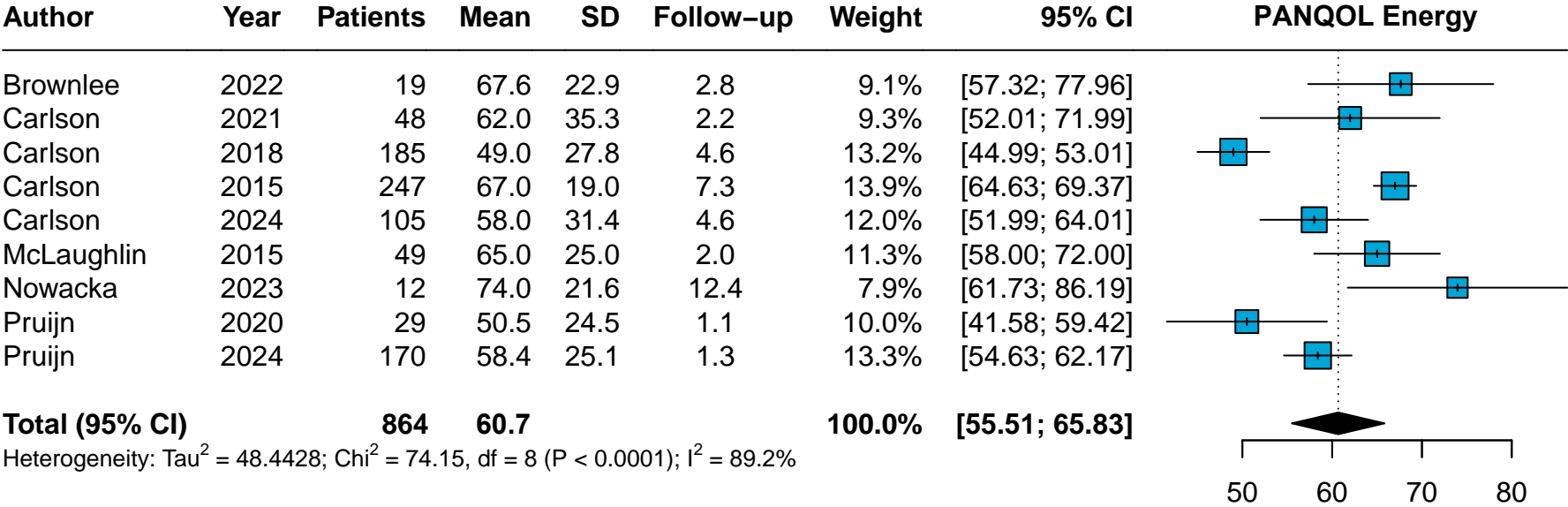

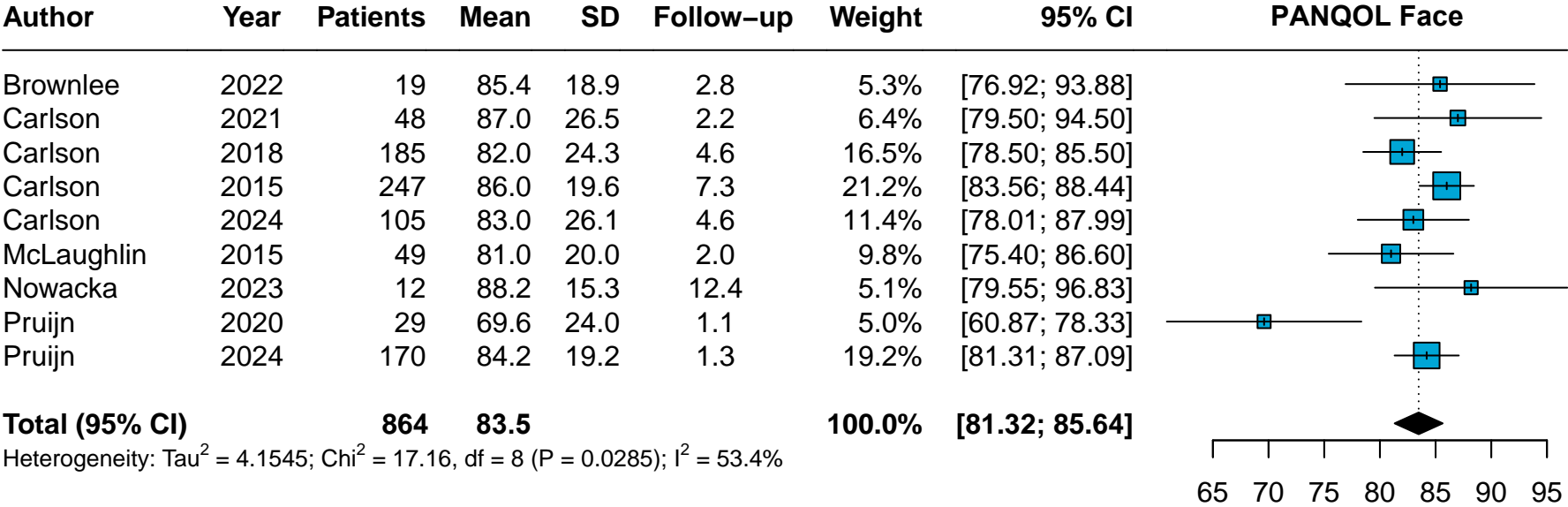

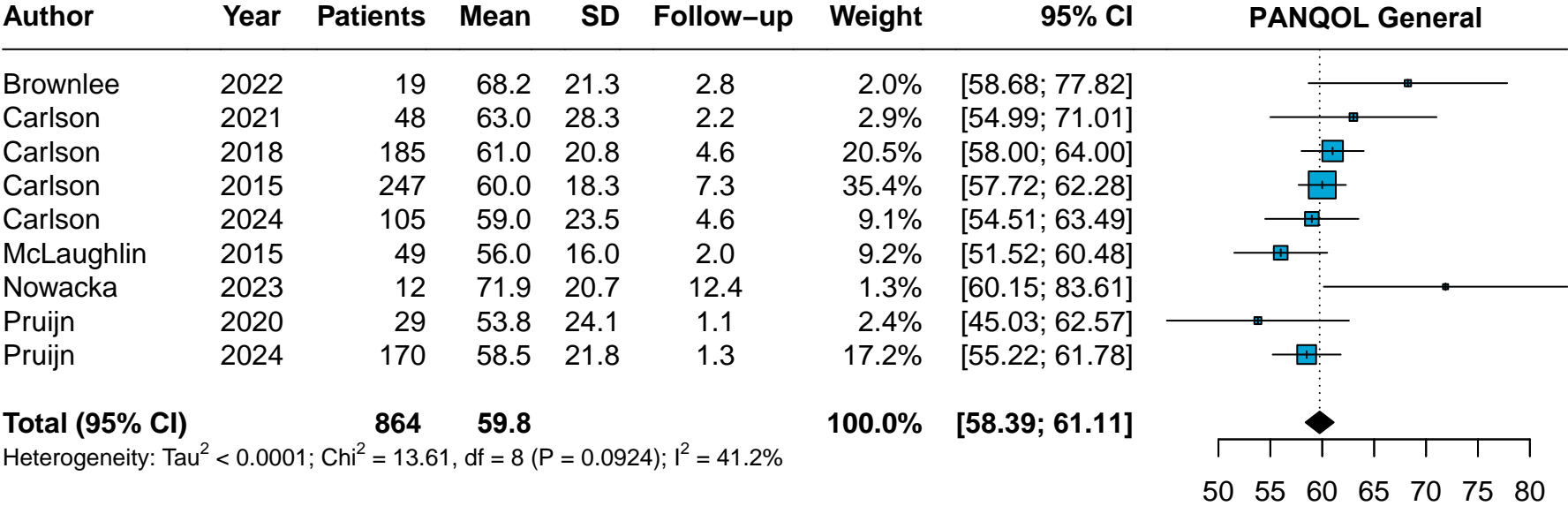

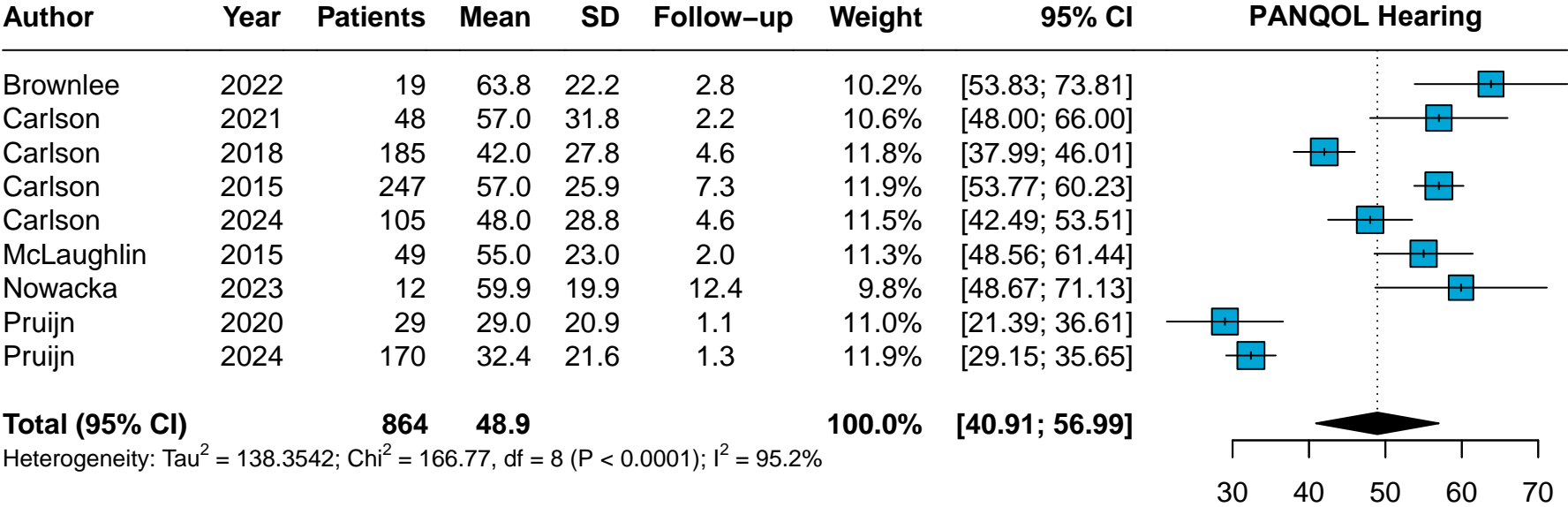

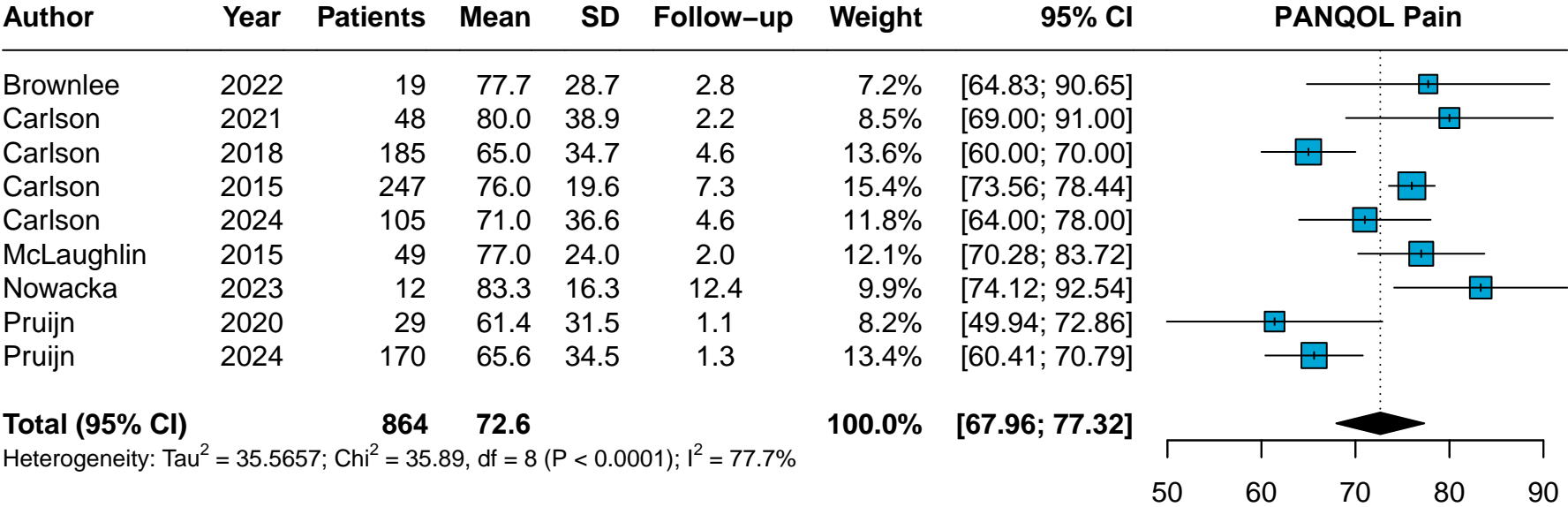

Supplement: Supplementary file 6 — Supplementary file6 (PDF 102 kb) [file 415_2026_13730_MOESM6_ESM.pdf]

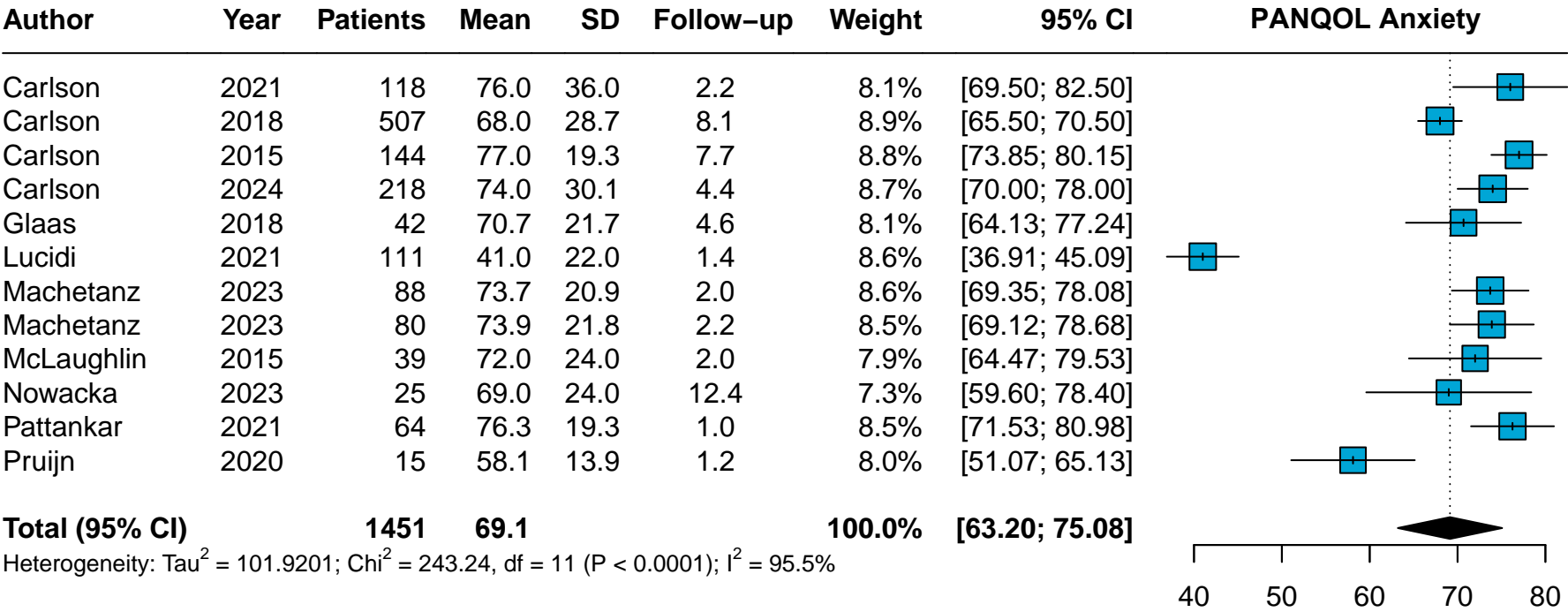

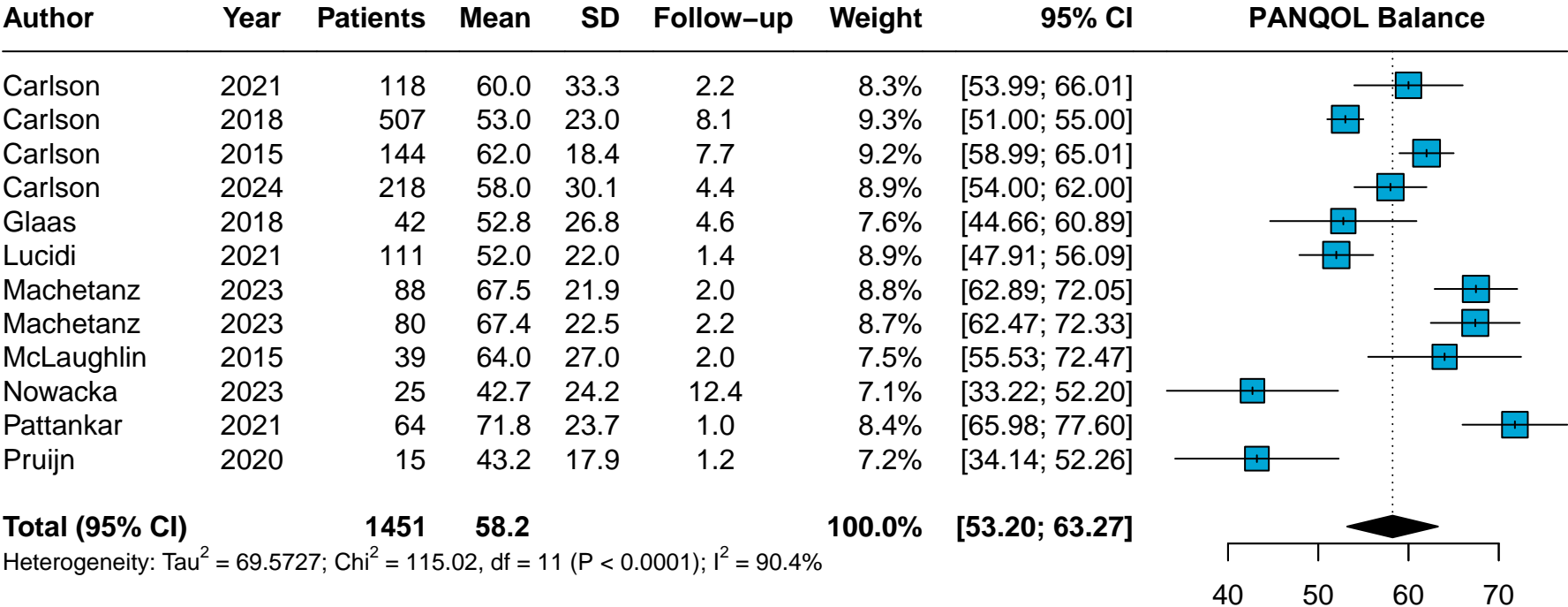

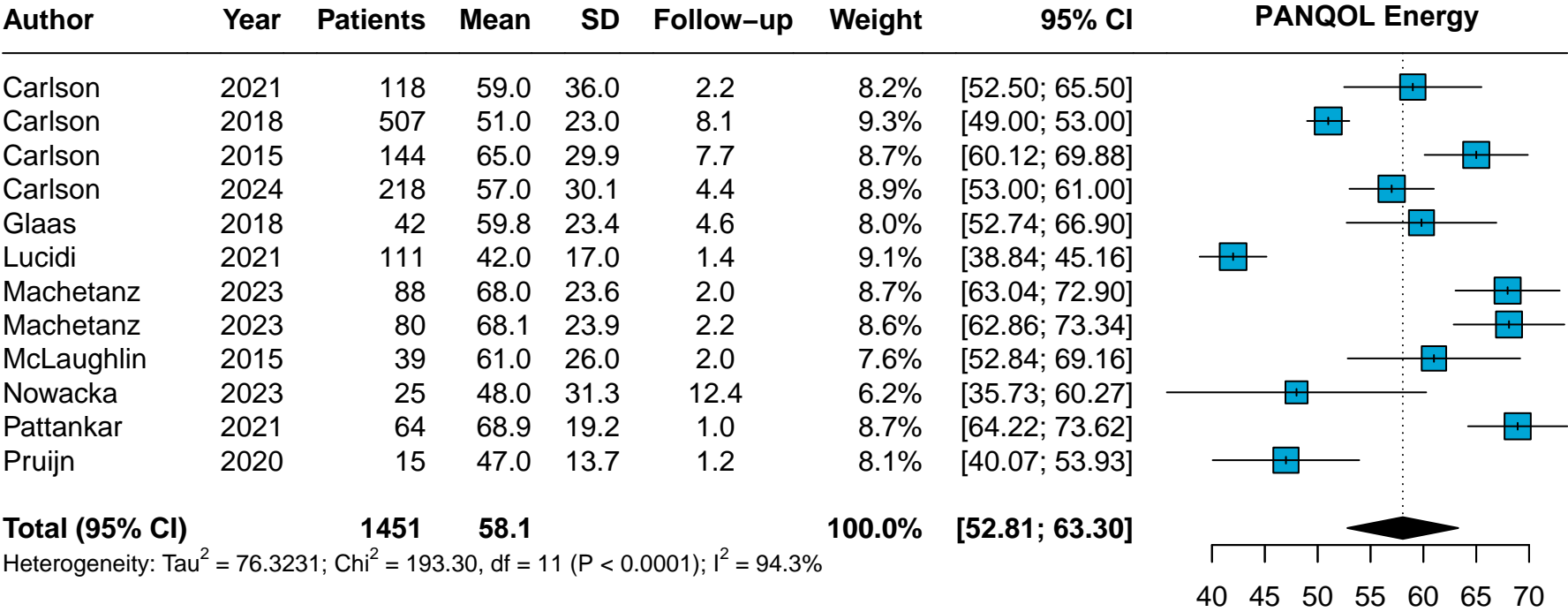

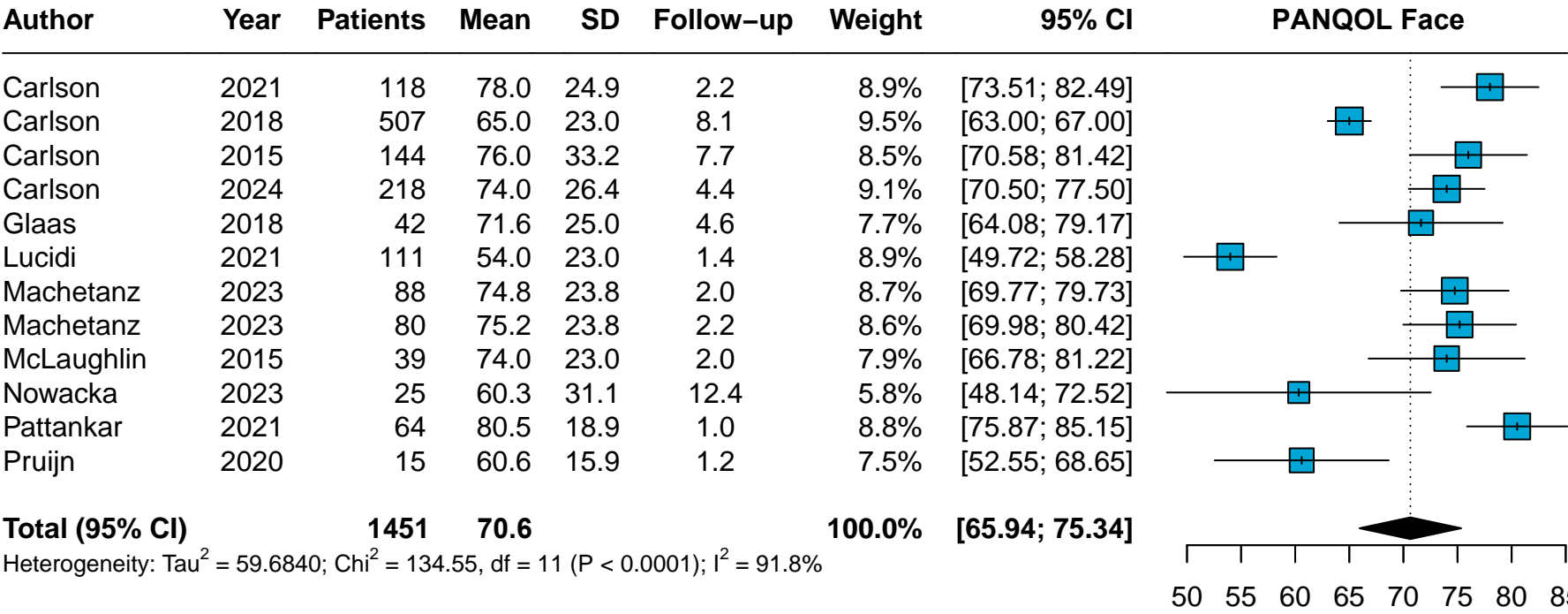

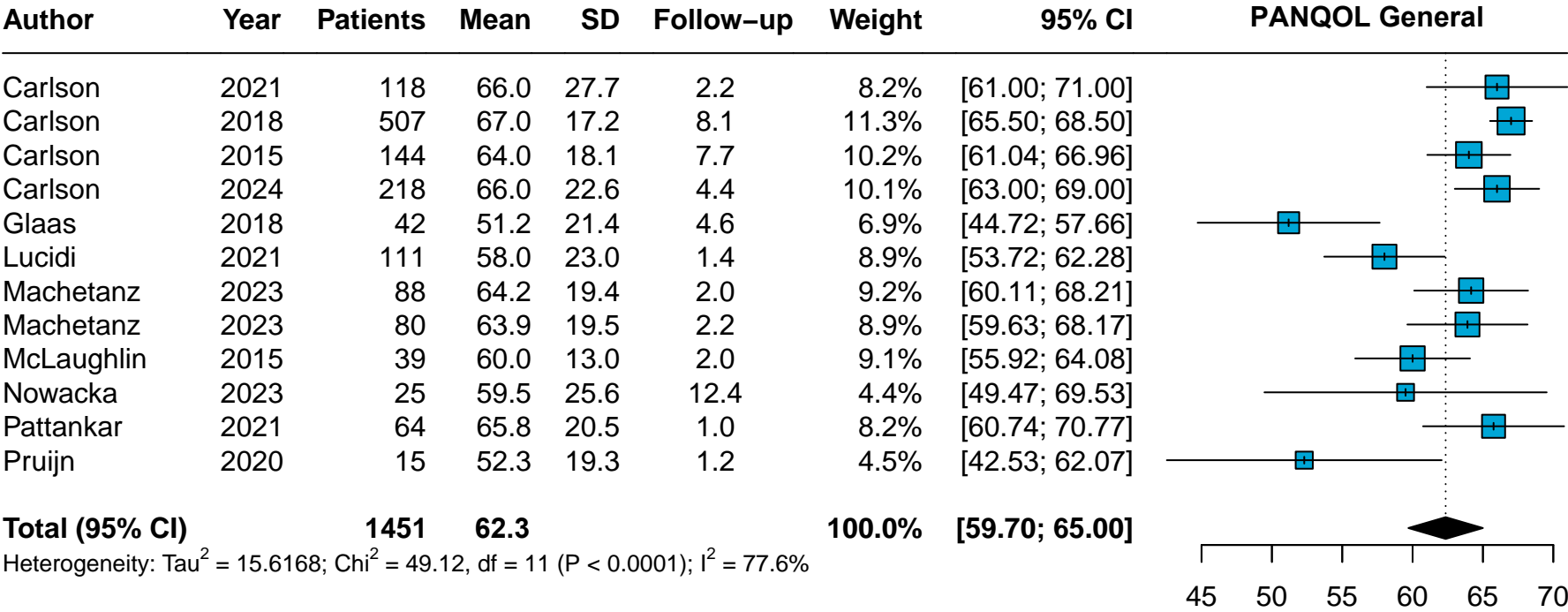

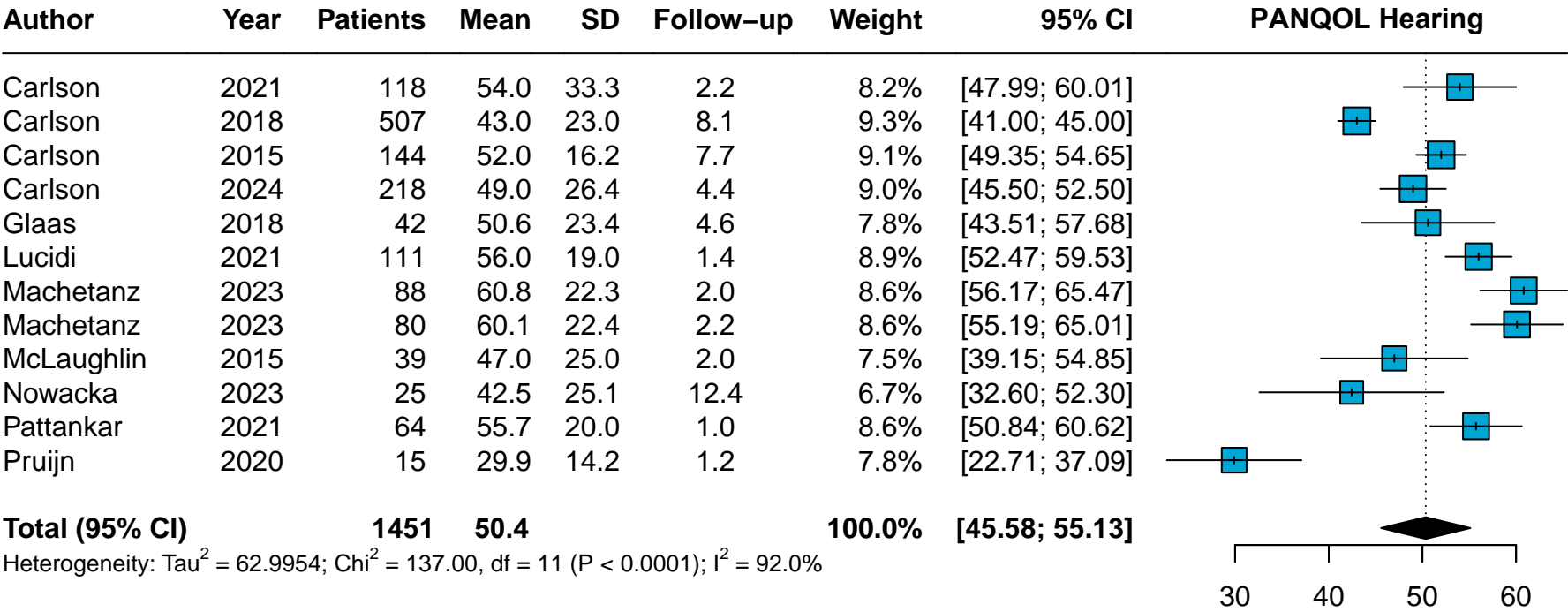

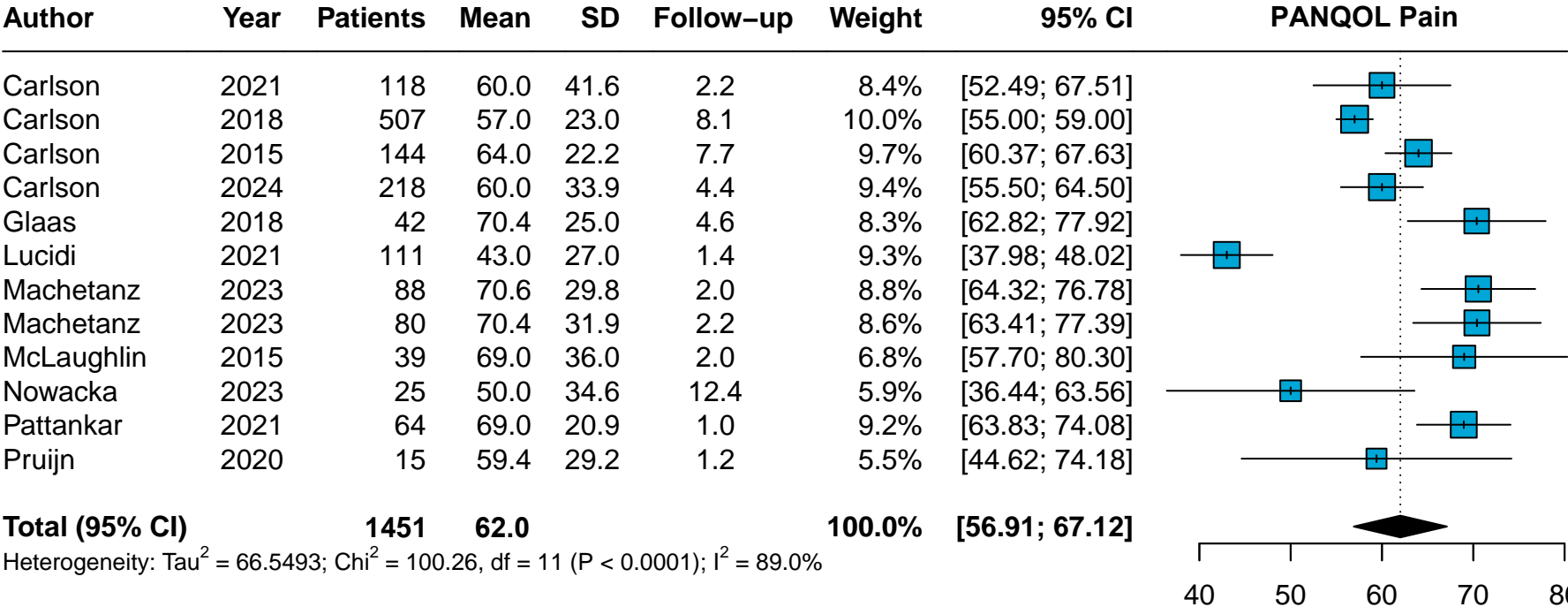

Supplement: Supplementary file 7 — Supplementary file7 (PDF 140 kb) [file 415_2026_13730_MOESM7_ESM.pdf]
